# Supplementary material for: NKX2-1 drives neuroendocrine transdifferentiation of prostate cancer via epigenetic and 3D chromatin remodeling
Source: Nat Genet. 2025 Jul 21;57(8):1966–80. doi: 10.1038/s41588-025-02265-4 (PMC12339387; doi:10.1038/s41588-025-02265-4)
Supplement: Supplementary file 1 — Supplementary Notes 1–5, Supplementary Methods 1–12 and Supplementary Figs. 1 and 2. [file 41588_2025_2265_MOESM1_ESM.pdf]

nature genetics

Supplementary information https://doi.org/10.1038/s41588-025-02265-4

NKX2-1 drives neuroendocrine transdifferentiation of prostate cancer via epigenetic and 3D chromatin remodeling

In the format provided by the authors and unedited

Table of Contents

Supplementary Notes
Supplementary Note 1: FOXA2 overexpression drives NET of PCa cells with clinically relevant phenotypes and molecular alterations
Supplementary Note 2: Epigenetic remodeling during NET of PCa
Supplementary Note 3: Clonal variance analyses using single-cell RNA-seq data
Supplementary Note 4: NKX2-1 is directly induced by ASCL1 and cooperates with ASCL1 to drive NET
Supplementary Note 5: CBP/p300 regulates lineage-specific super-enhancers (SEs)

Supplementary Methods
Supplementary Method 1: Lentivirus generation and infection
Supplementary Method 2: Co-immunoprecipitation (Co-IP)
Supplementary Method 3: 3D organoid cell culture
Supplementary Method 4: Colony formation assay
Supplementary Method 5: Mass spectrometry analysis
Supplementary Method 6: RNA extraction, RT–qPCR, and RNA-seq
Supplementary Method 7: ChIP, ChIP–seq, and ATAC–seq
Supplementary Method 8: ChIP–seq, super-enhancer (SE) and ATAC-seq analyses
Supplementary Method 9: Quantification of *FOXA2* and *NKX2-1* double-positive cells in D21, as well as the integrative analysis of LNCaP+FOXA2 scRNA-seq data with human PCa
Supplementary Method 10: Single-nucleotide variant (SNV) and germline mutation analyses in LNCaP+FOXA2 time-course cells
Supplementary Method 11: DiMeLo-seq sample preparation and analysis
Supplementary Method 12: Immunohistochemistry (IHC)

Supplementary Figures
Supplementary Figure 1. GO analyses of genes linked to luminal loops and NE loops
Supplementary Figure 2. CCS1477 targets lineage-specific enhancers active in a particular cell

Supplementary References

Supplementary Notes

Supplementary Note 1: FOXA2 overexpression drives NET of PCa cells with clinically relevant phenotypes and molecular alterations

We infected LNCaP (adenocarcinoma) cells with FOXA2 lentivirus and monitored the cells over the course of 28 days. Interestingly, approximately 30% of FOXA2-overexpressing cells (LNCaP+FOXA2) exhibited NE phenotype with neurite extensions and formed "cluster" morphology at day 21 (D21), which increased to over 90% at D28 (**Extended Data Fig. 2a**). Similarly, FOXA2 overexpression (OE) led to NET of 22Rv1 cells over a period of 28 days and of the AR-negative DU145 cells in only 7 days (**Extended Data Fig. 2b**). On the flip side, FOXA2 is up-regulated in a previously reported NEPC model of LNCaP with *TP53* and *RB1* KD [1], and FOXA2 knockdown (KD) abolished NE marker gene expression (**Extended Data Fig. 2c**). Being consistent with NET, LNCaP+FOXA2 cells formed much more rapidly growing xenograft tumors than the control cells (plv), with mixed histology varying from adenocarcinoma to small-cell carcinoma (**Extended Data Fig. 2d-f**), which is frequently observed in treatment-induced NEPC in patients. Of note, most (76.9%) FOXA2-positive tumor cells also expressed SYP, supporting NET (**Extended Data Fig. 2d-f**).

RNA-seq analyses of time-course LNCaP+FOXA2 cells revealed a large number of genes (n=12,835, DESeq2 likelihood ratio test, adjust p<0.0001) that were differentially expressed, indicating major transcriptional reprogramming. K-means clustering revealed 6 clusters, including 3 clusters each of up-regulated (C1-C3, n=6,825) and down-regulated genes (C4-6, n=6,010) (**Fig. 2a**). GO analyses showed that the biggest gene clusters, C2 and C5, are respectively induced genes involved in neurogenesis and embryonic development (e.g., *SOX2* and *POU3F2*) and repressed genes essential for luminal cell functions (e.g., *HOXB13* and *AR*), with the molecular switch occurring between D14 and D21. Early-response clusters of genes (C1 and C4) increased DNA damage response and decreased rRNA processing that occurred between D2 and D7, indicating cellular stress. Interestingly, the cells might have coped with cellular stress by transiently reducing mRNA metabolism (C3) and increasing lipid biosynthesis (C6) until they reach a stable NE-transformed state.

To explore whether the 3D chromatin organization is rewired during FOXA2-induced NET, we performed in situ Hi-C of time-course LNCaP+FOXA2 cells and several additional PDX tumors. First, hierarchical clustering of the pairwise correlation coefficients of Hi-C data using their PC1 matrices demonstrated a tight clustering of D0 and D14 cells with CRPC tumors, except LuCaP147, which reportedly has an intermediate transcriptome [2] (**Extended Data Fig. 2g**). Interestingly, despite the NE-like transcriptome, D21 cells remained in the CRPC cluster, suggesting a lag in stable chromatin re-organization during NET. The D28 cells were further distant from the CRPC cluster but yet insufficient to join the NEPC group, which may be partially attributed to the much higher heterogeneity in chromatin compartmentalization of the NEPC tumors. Furthermore, to examine the chromatin architecture during NET at the loop level, we identified 3,094 luminal loops (loops enriched in luminal cells) and 1,884 NE loops (loops enriched in NE cells) by comparing D0 with D28. As expected, the genes potentially regulated by NE loops were implicated in axonogenesis and developmental processes, and the genes controlled by luminal loops were involved in epithelial functions (**Supplementary Fig. 1a**).

APA analyses demonstrated that the luminal loops were gradually attenuated from D0 to D28, while the NE loops were strengthened over time (**Fig. 2d**). Interestingly, the APA scores of D28 cells were comparable to those of NCI-H660, supporting a re-organized 3D chromatin

architecture that is similar to prototype NEPC cells. Therefore, our data demonstrate that the distinct 3D chromatin architecture observed in patient-derived CRPC and NEPC tumors can be recapitulated in vitro in isogenic cells transforming from luminal to NE lineage.

Supplementary Note 2: Epigenetic remodeling during NET of PCa

To understand the epigenetic bases of the transcriptional reprogramming and 3D chromatin re-organization during NET, we performed Assay for Transposase-Accessible Chromatin using sequencing (ATAC-seq) experiment in time-course LNCaP+FOXA2 cells and identified 89,636 chromatin regions with significantly (adjusted p-value<0.0001) altered accessibility. PCA analyses showed that D0/2/7 cells and D21/28 cells were respectively clustered closer to CRPC and NEPC PDX tumors [3], whereas the D14 cells were positioned somewhere in between, indicating a transitioning stage (**Extended Data Fig. 3a**). K-means clustering of ATAC-seq peaks revealed 6 major clusters, including 2 clusters (C1-C2) of peaks that are shared over all timepoints, 2 clusters (C3-C4) of gradually increasing peaks, and 2 clusters (C5-C6) of gradually decreasing peaks (**Fig. 3a and Extended Data Fig. 3b**). The C3-C4 peaks became detectable only at D14 and localized primarily at enhancer elements, hereafter defined as NE enhancers (**Fig. 3b**). Motif analyses indeed identified neuronal and stem cell TFs, such as NKX2-1, and SOX2, as the top enriched TFs at these regions (**Fig.3a and Extended Data Fig. 3c**). Furthermore, GO analyses revealed that the genes within 50kb of these peaks were involved in neuron fate specification and stem cell development/function. On the contrary, C5-C6 peaks were strong in D0-D7 luminal cells and became inaccessible over time, thus defined as luminal enhancers (**Fig. 3a,b**). These peaks were enriched for motifs of luminal TFs such as FOXA1, AR, and HOXB13 and corresponded to genes involved in prostate luminal cell functions. The shared peaks, especially C1, were predominantly at promoter regions and enriched for motifs of basic TFs, such as SP1, likely critical for housekeeping gene expression. Altogether, these results support an evident chromatin remodeling that is consistent with the luminal-to-NE lineage switch from D0 to D28 cells and resembles clinical CRPC/NEPC samples.

Next, we exploited single-cell (sc) multiome (scRNA-seq and scATAC-seq) to capture individual, transitioning cells with intermediate transcriptome and chromatin states. We captured 2,110 D0, 1,739 D14, and 2,344 D21 LNCaP+FOXA2 cells from scMultiome analyses. First, comparison of the transcriptome status of our cells with clinical PCa cells [4] revealed that the majority of our D0 cells were clustered in close proximity to primary PCa cells, most of the D14 and some of the D21 cells were grouped along with CRPC cells, while a majority of D21 cells were in the neighborhood of NEPC cells (**Extended Data Fig. 3d**). Trajectory analyses further validated that our D14 cells and patient CRPC cells were in the middle stage of the pseudotime (**Extended Data Fig. 3e**). UMAP analyses of our scRNA-seq data revealed two distinct clusters that comprised primarily of D0 and D21 cells a (**Fig. 3c and Extended Data Fig. 3f**). Of note, most of the D14 and some of the D21 cells still belonged to the D0 cluster. These cells remained *AR*-positive but had a reduced AR signature (ARS) score and started to express some NE signature (NES) genes (**Fig. 3d-f**), indicating an intermediate transcriptome. To investigate this more closely, we quantified the proportion of cells at each timepoint that express *AR*, ARS, or NES genes and found that a majority (96.5%) of D0 cells were *AR*+/ARS+ (**Fig. 3f**), as expected for androgen-dependent PCa. Critically, this number dropped to around 55% at D14 with the emergence of a new cell type (~30%) that remained *AR*+ but had lost ARS expression (*AR*+/ARS-). *AR* itself was ultimately depleted in most (~65%) of the D21 cells, a majority of which also gained the expression of NES genes (*AR*-/NES+).

Likewise, we performed UMAP analyses of matched scATAC-seq data and also found two distinct clusters (**Fig. 3h**). Focusing on chromatin accessibility at the promoter and intragenic regions of AR and NE signature genes, we found that the chromatin of AR signature genes was accessible mostly in D0 cells, whereas that of NE signature genes was much more accessible in D21 cells, as expected (**Fig. 3i**), with concordant changes in signature gene expression (**Fig. 3j**). Importantly, although most D14 cells clustered together with D0 cells, many D14 cells already showed inaccessible chromatin at AR signature genes and modest chromatin accessibility at NE genes, similar to the D21 cells within the cluster (**Fig. 3i**), suggesting an intermediate chromatin state undergoing transformation.

Supplementary Note 3: Clonal variance analyses using single-cell RNA-seq data
As cancer cell lines, such as LNCaP, have been shown to contain multiple clones, which undergo varying copy number evolution during stress [5-7], we attempted to examine the clonal behaviors of LNCaP cells over NET. Single-cell Copy Number Variation (CNV) estimated from the scRNA-seq data using CopyKAT [8] identified two major clones: clone1 (luminal) dominating D0/D14 cells and decreasing over time, and clone2 (NE) increasing over time and dominating D21 cells (**Extended Data Fig. 4a,b**). Critically, cells of both clonality were ARS+/NES- at D0, and showed a clear reduction in ARS but remained NES- at D14, indicating a transformation of both clones (**Extended Data Fig. 4c**). By D21, clone1 cells remained ARS-intermediate/NES-, but clone2 cells completely lost ARS and became NES+. These findings were further validated by single-cell nanopore RNA sequencing (scNanoRNA-seq) [9], which provided additional information on germline mutations that supported the two clones as isogenic subclones of LNCaP cells (**Extended Data Fig. 4d-h**). In conclusion, cells of both clonality types went through NET from D0 to D21, with clone2 being fully transformed and becoming the dominant clone at D21.

To validate that the two clones are isogenic, we analyzed D0 and endpoint D28 LNCaP+FOXA2 cells using single-cell nanopore RNA sequencing (scNanoRNA-seq) [9], which captures the full-length mRNAs and/or pre-mRNAs, thus providing a deeper and broader read coverage. We identified 1093 Single-Nucleotide Variants (SNVs) that were mutated in at least 5% of high-quality D0 and D28 cells, which, likewise, clustered the cells into two clone types: clone1 predominantly at D0, while clone2 dominant at D28 (**Extended Data Fig. 4d-e**). Again, despite their different clonality, both clone1 and clone2 D0 cells were ARS+/NES- and became ARS-/NES+ in D28 cells (**Extended Data Fig. 4f-g**), suggesting that cells of both clonality types went through initial NE transformation, further supporting a mixed model of cell transformation and clonal selection. Next, we analyzed germline mutations, defined as SNVs that were mutated in more than 95% of cells with detection in each timepoint, and found 585 and 357 germline mutations in D0 and D28 cells, respectively. Critically, a significant 139 germline mutations ($p<0.001$, Fisher's exact test) were shared between D0 and D28 cells (**Extended Data Fig. 4h**). In addition, the rest also showed an overall trend of consistency, despite many of them being detected in only one but not the other timepoint, wherein they had zero or insufficient read coverage to capture the mutant allele. Altogether, these results strongly support that the D0 and D28 cells were isogenic subclones of LNCaP cells.

Supplementary Note 4: NKX2-1 is directly induced by ASCL1 and cooperates with ASCL1 to drive NET

NKX2-1 was ubiquitously up-regulated in NEPC, even in some FOXA2-low tumors, indicating additional upstream regulators (**Fig. 5b-c**). We found that *ASCL1* expression was strongly correlated with *NKX2-1* in PDX tumors (**Extended Data Fig. 7a,b**). Further, ASCL1 bound to the *NKX2-1* gene to directly induce its expression (**Extended Data Fig. 7c,d**). Moreover, NKX2-1 co-occupied some ASCL1-binding sites, and depleting either of them attenuated NEPC cell growth (**Extended Data Fig. 7e,f**), in agreement with a previous report of NKX2-1 in facilitating ASCL1 in mediating NEPC[10]. Taken together, these results suggest *NKX2-1* gene transcription was induced in NEPC cells largely due to TF binding and epigenetic regulation and that NKX2-1 could cooperate with multiple co-factors, including FOXA2 and ASCL1, to induce NET of PCa.

Supplementary Note 5: CBP/p300 regulates lineage-specific super-enhancers (SEs)
Although p300/CBP has been shown to critically regulate AR, c-Myc, and luminal enhancers and are thus critical therapeutic targets in CRPC [11,12], they have not been well studied in NEPC. We found that similar to p300, H3K27ac was strongly enriched at D28 FOXA2 binding sites (NE enhancers) in the LuNE cells, which were eliminated by CCS1477 (**Fig. 7d**). By contrast, H3K27ac marked luminal enhancers in LNCaP cells, which were also depleted by CCS1477 treatment (**Extended Data Fig. 9b**). This data indicates that cells of different lineages harbor a distinct set of active enhancers marked by H3K27ac, and by inhibiting H3K27ac, CCS1477 may selectively target lineage-specific enhancers, many of which are known as super-enhancers (**SE**) of the cancer type. To test this, we performed SE analyses using H3K27ac ChIP-seq data by ROSE [13] and identified 394 SEs in LuNE cells. In agreement with the previous notion that SEs control lineage-specific TFs and proto-oncogenes [14], SE-associated genes in LuNE cells include embryo/neuron-specific TFs, such as HOXA1, HOXB2, and ZIC5 [15-17], and proto-oncogenes, such as SOX2 and NFIB that have been implicated in NEPC and small-cell lung cancer [1,18-20] (**Extended Data Fig. 9c**). Notably, CCS1477 treatment of LuNE cells markedly reduced H3K27ac at these SEs (**Extended Data Fig. 9d**). By contrast, genes associated with SEs in LNCaP cells are prostatic TFs, such as HOXB13, FOXA1, and GATA2, and proto-oncogenes, such as CCND1 and MYC, and CCS1477 inhibited these SEs in LNCaP cells (**Extended Data Fig. 9e,f**).

GO analysis of CCS1477-repressed genes in LuNE cells enriched in neurogenesis and embryonic development processes (**Fig.7g**), whereas, in LNCaP cells, CCS1477 suppressed a distinct set of genes that are enriched in hormonal response (**Extended Data Fig. 9k**). These differences may be due to CCS1477 targeting lineage-specific SEs. Indeed, GSEA analyses showed that distinct sets of SE-associated genes in LNCaP and LuNE cells were suppressed by CCS1477 treatment of LNCaP and LuNE cells, respectively (**Fig. 7h** and **Supplementary Fig.2a,b**). In addition, colony formation assays have shown that CCS1477 inhibits the growth of LNCaP and LuNE, but not benign RWPE-1 and BPH-1 prostate cells, further supporting CCS1477 targeting of lineage-specific SEs to which many cancer cells are addicted.

Supplementary Methods

Supplementary Method 1: Lentivirus generation and infection

For the generation of lentivirus, HEK293T cells were transfected with psPAX2 and pMD2G with the target gene at a ratio of 2:1:1. The supernatant containing lentiviruses was harvested at 48 h after transfection and filtered through a 0.45μm filter. Lentiviruses, supplemented with 8μg/mL polybrene, were used to infect human PCa cells. For most knockdown experiments with shRNAs or gRNAs, cells were harvested on day 5 after infection. For organoid infection, the single cells were mixed with lentivirus-containing polybrene with a final concentration of 8μg/mL, then centrifuged for 1 h at 600g at room temperature. Cells were subsequently placed at 37°C, 5% CO_2, for 6 h to recover before being plated in Matrigel.

Supplementary Method 2: Co-immunoprecipitation (Co-IP)

Nuclear fraction was used for all Co-IP experiments in this study. Nuclear proteins were isolated as previously with some modifications [21,22]. Briefly, cells were resuspended in buffer A (10 mM HEPES, pH 7.9, 10 mM KCl, 1.5 mM $MgCl_2$, 0.34 M sucrose, 10% glycerol, 1 mM dithiothreitol, 1mM EDTA, 1 × Roche protease inhibitor cocktail) and incubated on ice for 10 min. Then, the TritonX-100 with a final concentration at 0.1% was added to the cell suspension to extract cytoplasmic fraction. After washing once with buffer A, the nuclei were incubated in buffer B (10mM HEPES, pH7.9, 10% glycerol, 1 mM EDTA, 1.5 mM $MgCl_2$, 300 mM NaCl, 0.5% NP 40, 1 mM dithiothreitol, 1 × Roche protease inhibitor cocktail) for 30 min on ice to isolate the nuclear fraction. The nuclear fraction was first pre-cleared with protein G or A-magnetic beads at 4 °C for 2 h, followed by incubation with the corresponding antibody overnight. Dynabeads protein G beads (Life Technologies) were added the next day and incubated for 1 h at 4 °C. The beads/protein complex were washed four times with IP wash buffer (10mM HEPES, pH7.9, 1 mM EDTA, 1.5 mM $MgCl_2$, 150 mM NaCl, 0.5% NP 40, 1 mM dithiothreitol, 1 × Roche protease inhibitor cocktail) and eluted with 30 μl of 2 × SDS sample buffer and subjected to western blot (WB) analysis using the corresponding antibodies.

Supplementary Method 3: 3D Organoid cell culture

LuCaP145.2 and LuCaP93 PDX-derived organoids were generated as previously described[23]. Briefly, LuCaP145.2 or LuCaP93 tumors were minced into small pieces (∼1 mm^3) and digested in 5 mg/ml collagenase type II (Gibco, Cat#17101-015) with 10 μM Y-27632 dihydrochloride (Tocris, Cat#1254) in a 15-ml Falcon tube for 1–1.5 h at 37 °C. Digested tissues were washed once with Advanced DMEM/F12 medium containing penicillin/streptomycin, 10 mM HEPES and 2 mM GlutaMAX (adDMEM/F12 +/+/+), then resuspended in 5 mL of TrypLE Express (Gibco, 12604-021), and further digested at 37 °C for 15 min. After digestion, cells were washed once with adDMEM/F12 +/+/+ and counted with a hemocytometer. A total of 20,000 cells were mixed with 50% Matrigel and plated in a 12-well tissue culture plate, and placed in a CO_2 incubator (5% CO_2, 37 °C) for 15 min to allow Matrigel to solidify. Pre-warmed human complete organoids medium plus 10 μM of Y-27632 dihydrochloride was gently added to cells, and cells were maintained in a CO_2 incubator (5% CO_2, 37 °C). The medium was changed every 3 days, and the organoids were passaged biweekly at 1:3.

Supplementary Method 4: Colony formation assay

For colony formation assay, LuNE, and LNCaP cells with indicated gene alteration (5×10^3 cells per well), RWPE-1, and BPH-1 (2×10^3 cells per well) cells were seeded in 12-well plates. The cells were fixed by 4% paraformaldehyde after 2 weeks of growth and stained with 0.05% crystal violet. The colonies were imaged with ChemiDoc (BIO-RAD).

Supplementary Method 5: Mass spectrometry analysis
Chromatin fraction was used for the mass spectrometry experiments in this study. Chromatin proteins were isolated as previously with some modifications [21,22]. Briefly, cells were resuspended in buffer A (10 mM HEPES, pH 7.9, 10 mM KCl, 1.5 mM MgCl2, 0.34 M sucrose, 10% glycerol, 1 mM dithiothreitol, 1 × Roche protease inhibitor cocktail) and incubated on ice for 10 min. Then, the final concentration of 0.1% TritonX-100 was added to the cell suspension and vortexed for 15 s, and spun down at 4 °C for 5 min at 1,000g. The supernatant was kept as a cytoplasmic fraction. The nuclei pellet was washed once with buffer A, and then resuspended in buffer C (3 mM EDTA, 75 mM NaCl, 0.1% TritonX-100, 1 mM dithiothreitol, protease cocktail) for 30 min on ice to remove chromatin-free proteins. Insoluble chromatin was resuspended in buffer D (10mM HEPES, pH7.9, 10% glycerol, 1 mM EDTA, 1.5 mM MgCl2, 300 mM NaCl, 0.5% NP 40, 1 mM dithiothreitol, 0.5U/µl TurboNuclease (Accelagen, Cat#N0103M), 1 × Roche protease inhibitor cocktail) and incubated at 4 °C for 30 min in a rotor with 300 r.p.m. The chromatin was spun down for 10 min at 12,000g at 4 °C, and the supernatant was saved as the chromatin fraction. The chromatin fraction was first pre-cleared with protein A-magnetic beads at 4 °C for 2 h, followed by incubation with FOXA2 (Abcam, Cat#ab108422) or IgG (Sigma, cat#12-370) antibody overnight. Dynabeads Protein A beads (Life Technologies) were added the next day and incubated for 1 h at 4 °C. The beads/protein complex were washed four times with IP wash buffer (10mM HEPES, pH7.9, 1 mM EDTA, 1.5 mM MgCl2, 150 mM NaCl, 0.5% NP 40, 1 mM dithiothreitol, 1 × Roche protease inhibitor cocktail) and eluted with 1xSDS sample buffer and subjected to SDS–PAGE. Protein bands were excised and subjected to mass spectrometry analysis using the Orbitrap Velos Pro system.

Supplementary Method 6: RNA extraction, RT–qPCR, and RNA-seq
RNA extraction, RT–qPCR, and RNA-seq were performed as previously [21] with some modifications. RNA was extracted using the nucleospin RNA kit (Takara) according to the manufacturer's recommended protocol. Then, 500 ng of RNA was reverse transcribed into complementary DNA (cDNA) using the ReverTra Ace qPCR RT Master Mix kit (Diagnocine) according to the manufacturer's recommended protocol. qPCR was performed with 2X Universal SYBR Green Fast qPCR Mix (Abclonal, Cat#RK21203) and QuantStudio 3 (Thermo Fisher). For RNA-seq, total RNA was isolated as described above and performed in triplicate. RNA-seq libraries were prepared from 0.5 µg of high-quality DNA-free RNA using NEBNext Ultra RNA Library Prep Kit, according to the manufacturer's instructions. The libraries passing quality control (equal size distribution between 250 and 400 bp, no adapter contamination peaks, no degradation peaks) were quantified using the Library Quantification Kit from Illumina (Kapa Biosystems, KK4603). Libraries were pooled to a final concentration of 10 nM and sequenced paired-end using the Illumina NovaSeq 6000.

Supplementary Method 7: ChIP, ChIP–seq, and ATAC–seq
ChIP, ChIP–seq were performed using the previously described protocol with the following modifications [21,22]. For FOXA2, NKX2-1, H3K27ac, and H3K4me1 ChIP, PCa cells were

crosslinked with 1% formaldehyde (Thermo Fisher, Cat#28908)) for 10 min at room temperature with gentle rotation and then quenched for 5 min with 0.125 M glycine. 10 million cells were used for each FOXA2, NKX2-1 ChIP, and 5 million cells were used for each H3K4me1, H3K27ac ChIP. Chromatin was sonicated to an average length of 200–600 bp using an E220 focused ultrasonicator (Covaris). Supernatants containing chromatin fragments were pre-cleared with protein A agarose beads (Millipore) for 40 min and incubated with a primary antibody overnight at 4 °C on a rotator. 50 µl of protein A agarose beads were added at the next day and incubated for 2 h at 4 °C. Beads were washed twice with 1x dialysis buffer (2 mM EDTA, 50 mM Tris-Cl, pH 8.0) and four times with ChIP wash buffer (100 mM Tris-Cl, pH 9.0, 500 mM LiCl, 1% NP40, 1% deoxycholate). The antibody/protein/DNA complex were eluted with elution buffer (50 mM NaHCO3, 1% SDS), the crosslinks were reversed, and DNA was purified with DNA Clean & Concentrator-5 kit (ZYMO Research). For H3K27ac ChIP in *p300*, *CBP*, *NKX2-1*, *FOXA2*-KD or CCS1477-treated cells, Drosophila S2 spike-in normalization was performed following the protocol from Active Motif. ChIP–seq libraries were prepared from 3–5 ng of ChIPed DNA using NEBNext Ultra II DNA Library Prep Kit (NEB, E7645S), according to the manufacturer's instructions. Post-PCR libraries were size selected between 250 and 450 bp using Agencourt AMPure XP beads from Beckman Coulter and were quantified using the Library Quantification Kit from Illumina (Kapa Biosystems, KK4603). Libraries were pooled to a final concentration of 10 nM and sequenced single-end using the Illumina HiSeq 4000 or NovaSeq 6000.

For FOXA2, NKX2-1, H3K4me1, H3K4me3 and H3K27ac ChIP in LuCaP PDX, double crosslinking was performed. 50 mg of homogenized LuCaP PDX tumor tissues were crosslinked with 2 mM Disuccinimidyl glutarate (DSG, Pierce) for 10 min at room temperature, followed by 1% formaldehyde for 10 min. Crosslinked cells were then quenched with 0.125 M glycine for 5 min at room temperature. Chromatin shearing, immunoprecipitation, and library preparation were performed as described above.

The assay for transposase-accessible chromatin using sequencing (ATAC–seq) was performed as previously reported [24]. Briefly, 50,000 cells were washed once with 1 × PBS and resuspended in 100 µl of lysis buffer (10mM Tris-HCl, pH 7.4, 10mM NaCl, 3mM MgCl2, 0.1% NP 40, 0.1% Tween-20, 0.01% Digitonin) for 3min on ice, followed by adding 1ml of wash buffer (10mM Tris-HCl, pH 7.4, 10mM NaCl, 3mM MgCl2, 0.1% Tween-20) to remove cytoplasm and mitochondria fraction. The nuclei pellet was resuspended in 25 µl transposition mix (12.5 µl of 2x TD buffer, 2.5 µl of Tn5 Transposome, 8.25 µl of 1xPBS, 0.25 µl of 1% digitonin, 0.25 µl of 10% Tween-20, 1.25 µl H2O) and incubated at 37 °C for 30 min in a thermomixer with 300 r.p.m. The tagmented DNA was purified using the DNA Clean & Concentrator-5 kit (ZYMO Research). Libraries were amplified, and adapter dimers and primer dimers were cleaned up. The libraries were sequenced paired-end using an Illumina HiSeq 4000.

Supplementary Method 8: ChIP–seq, super-enhancer (SE) and ATAC-seq analyses
ChIP–seq reads were aligned to the Human Reference Genome (assembly hg19) using Bowtie2 2.0.5. FastQC (0.12) was used to check quality control. The adapter was trimmed by Trim Galore (0.6.5). Non-uniquely mapping and redundant reads, and blacklists were removed. The remaining reads were used to generate binding peaks with MACS2 with a q-value (FDR) threshold of 0.01. For replicates, the peaks were merged for downstream analysis. Weighted Venn diagrams were created by the R package Vennerable (3.0). Heatmap views of ChIP-seq were generated by deepTools. Motif analyses were performed using HOMER. Genomic

distribution of ChIP-seq binding sites was generated by the R Bioconductor package ChIPseeker (1.36). For FOXA2 clusters in Fig.7d, the sample size was down sampled to the same, FOXA2 peaks with signal value greater than 10 were retained for generating the heatmap. For H3K27ac ChIP-seq analysis in Fig.7d and Extended Data Fig.9b, we used Drosophila S2 spike-in normalization strategy and followed the protocol from Active Motif.

Super-enhancers were identified by ROSE as previously reported [14]. Briefly, H3K27ac ChIP-seq peaks were stitched computationally if they were within 12,500 bp of each other, peaks within ± 2,000 bp from a RefSeq promoter were excluded from stitching. All the enhancers were ranked by H3K27ac signal. A clear inflection point in the visualized plot was used to separate SEs from all other enhancers. Enhancers with a H3K27ac signal above the inflection point were defined as SEs, and the left as typical enhancers (TEs). GREAT [25] was used to assign SE-associated genes.

For ATAC-seq analysis, the raw data were processed with the ENCODE ATAC-seq pipeline (1.8.0) (https://github.com/ENCODE-DCC/atac-seq-pipeline). In short, the reads were trimmed, filtered, and aligned against hg38 using Bowtie2. PCR duplicates, reads mapped to the mitochondrial chromosome or repeated regions were removed. To correct for the Tn5 transposase insertion, mapped reads were shifted +4/-5. Peak calling was performed using MACS2, with a p-value < 0.01 as the cutoff. Reproducible peaks from two biological replicates were defined as peaks with Irreproducibility Discovery Rates (IDR) < 0.05. A list of consensus peaks was created from non-overlap peaks based on summits extended by 250bp. Reads falling into consensus peaks were quantified by RSubread. For Fig.3a, differential ATAC-seq peaks were identified by DESeq2 using the Likelihood Ratio Test (LRT). Peaks were filtered with padj < 0.0001 and clustered using ComplexHeatmap k-means clustering.

Supplementary Method 9: Quantification of *FOXA2* and *NKX2-1* double-positive cells in D21, as well as the integrative analysis of LNCaP+FOXA2 scRNA-seq data with human PCa

For quantification of *FOXA2* and *NKX2-1* double positive cells in D21 due to the sparsity of scRNA-seq data, deeper sequencing data was used and modified methods were implemented. First, the ectopic sequences between the polyA tail and *FOXA2* stop codon were included in the reference GTF to ensure capture of ectopic FOXA2. Furthermore, Adaptively Thresholded Low-Rank Approximation (ALRA) was used to correct technical dropouts and resulted in an increased number of cells expressing *FOXA2* and *NKX2-1* using the RunALRA implementation in SeuratWrappers (0.3.0)[26] .

Patient samples' scRNA-seq data from Cheng et al.[4] was downloaded as bam files, converted into fastq files, and re-run using CellRanger (6.1.2). CellRanger aggr was run on the samples to recapitulate the original paper's pipeline. We assigned cell types based on the markers used in the paper. Our LNCaP+FOXA2 time-course and the patient samples' scRNA-seq data were normalized using SCTransform. Integration was performed using the SCTransform integration pipeline. After integration with the entire population, the non-epithelial cells and basal cells were removed, and a new UMAP was generated. The pseudotime was calculated with a node in the primary PCa population chosen as the starting cell.

Supplementary Method 10: Single-nucleotide variant (SNV) and germline mutation analyses in LNCaP+FOXA2 time-course cells

Transcriptome-wide gene expression and mutation calls of LNCaP+FOXA2 time-course cells (D0 and D28) were detected using single cell Nanopore sequencing analysis of Genotypes and Phenotypes Simultaneously (scNanoGPS) (v1.1) [9]. Low-quality cells were filtered based on percent of mitochondrial reads and the number of genes in each cell. The integrated UMAP and AR/NE signatures were created using LogNormalized expression. To accurately analyze transcriptome-wide single-nucleotide variants (SNVs) in single cells, SNV calls that were not supported by at least 2 reads containing the mutation were filtered out. In addition, to remove random errors, only mutations that occurred in more than 5% of high-quality cells in each condition were kept. Further, to preclude missed mutation calls due to undetectable gene expression in one timepoint but not the other, only mutations that were detected in both timepoints (D0 and D28) were selected for further analysis, resulting in 1,093 SNVs that passed all criteria. Variant Allele Frequency (VAF) was calculated for these mutations by dividing the read coverage supporting a mutation by the sum of all the reads detected for that nucleotide. Mutations were categorized into different types based on the value of VAF. In a nested conditional manner, if the mean of the VAF for a mutation across all cells was greater than or equal to 0.9, it is considered as mutated across all cells, if it is less than or equal to 0.3 it is considered as wild type (WT). If the mean VAF of a mutation in D28 is greater than or equal to 0.9, it's regarded as mutated only in D28, whereas if the mean VAF of a mutation in D0 is greater than 0.9, it's considered as WT in D28. Otherwise, it's considered mixed. These categories were used to order the rows in heatmap of Extended Data Fig.4d. Clones were called based on k-means clustering of VAF values with single imputation applied.

For the germline mutations, a similar analysis was performed, and germline mutations in each timepoint were called independently as previously reported [9]. SNVs that have read coverage (i.e. covered) in more than 50% of all cells of a particular timepoint and were mutated, supported by at least 1 mutant read, in more than 95% of these covered cells were defined as germline mutations. A total of 585 and 387 germline mutations were found in D0 and D28 cells, respectively. Further, mutations that have read coverage in one timepoint but not the other were removed, resulting in a total of 139 germline mutations detected in more than 50% of D0 and D28 cells.

Supplementary Method 11: DiMeLo-seq sample preparation and analysis

Directed methylation with long-read sequencing (DiMeLo-seq), a method that uses antibody-tethered enzymes to methylate DNA near a target protein's binding sites in situ, was performed using the previously described protocol with some modifications [27]. 2 million of D2, D14 and D28 LNCaP+FOXA2 cells or cells from 20mg PDX tumor tissues were used for each DiMeLo-seq. The cells were washed once with 1ml of 1xPBS, then cross-linked with 0.1% formaldehyde for 2 min at room temperature, followed by the addition of 75mM glycine to quench the reaction for 5 min at room temperature. Crosslinked cells were lysed in 1 ml of Dig-Wash buffer (0.02% digitonin, 20 mM HEPES-potassium hydroxide buffer, pH 7.5, 150 mM sodium chloride, 0.5 mM spermidine, 1 Roche cOmplete EDTA-free tablet (11873580001) per 50 ml buffer and 0.1% BSA) for 5 min on ice. The nuclei were bound to Concanavalin A-coated magnetic beads (BangsLaboratoriesBP531) for 10 min at room temperature. The beads-bound nuclei were resuspended in 200 µl of Tween-Wash buffer (0.1% Tween-20, 20 mM HEPES-potassium hydroxide, pH 7.5, 150 mM sodium chloride, 0.5 mM spermidine, 1 Roche cOmplete EDTA-free tablet per 50 ml buffer and 0.1% BSA) containing 4 µl FOXA2 antibody (Abcam, Cat#ab108422) or IgG (CST, Cat#66362), incubated on a rotator at 4 °C for overnight. After

primary antibody incubation, the beads were washed twice with 1 ml of Tween-Wash buffer, then beads-bound nuclei were gently resolved in 100 µl of Tween-Wash containing 200 nM pA–Hia5 and incubated on a rotator at 4 °C for 2h. After pA-Hia5 binding, the beads were washed twice with 1 ml of Tween-Wash buffer, followed by Hia5 methyltransferase reaction in 100 µl of Activation buffer (15 mM Tris, pH 8.0, 15 mM sodium chloride, 60 mM potassium chloride, 1 mM EDTA, pH 8.0, 0.5 mM EGTA, pH 8.0, 0.5 mM spermidine, 0.1% BSA and 800 µM SAM) and incubated at 37 °C for 2h with replenishing of 800 µM SAM during the incubation. After the incubation, the beads-bound nuclei were resuspended in 100 µl of clod PBS containing 2 µl of 20mg/ml Proteinase K, 1 µl of 10% SDS and incubated at 55 °C for 1h. The nanopore DNA sequence libraries were prepared as RRMS described above. The sequence metrics were updated in **Supplementary Table 5**. QC was performed by mosdepth (0.3.4) and NanoStat (1.6.0). Aggregated and modified base counts for 5mC and 6mA were performed by modbam2bed (0.9.5). Bigwig file of IGV track was generated by bedGraphToBigWig from kentUtils (302.1). A and CpG methylation profiles, intensity plots, heatmaps and single DNA molecule tracks were generated by dimelo (0.1.0) python package. Differentially methylated regions (DMRs) were identified by R Bioconductor package DSS (2.48.0). Top 200 hyper and top 200 hypo-DMRs-associated genes were annotated by HOMER (4.8.3) and generated in PCA using the Z-score of RPKM value of RNA-seq data.

Supplementary Method 12: Immunohistochemistry (IHC)

IHC was performed using ImmPRESS® Excel Amplified Polymer Kit (Vector Laboratories), according to the manufacturer's instructions. Briefly, Formalin-fixed paraffin-embedded (FFPE) tissue sections were de-paraffinized and hydrated, followed by antigen retrieval by heating at 99–100 °C in 1x citrate buffer, pH 6.0 (Sigma, C9999-1000ML) for 15 min. After antigen retrieval, the tissue sections were incubated with BLOXALL blocking solution to quench endogenous peroxidase activity, then blocked with 2.5% normal horse serum. After blocking, the tissue sections were incubated with primary antibodies, followed by incubating with an amplifier antibody (Goat Anti-Rabbit IgG for rabbit primary antibodies) or directly incubated with ImmPRESS polymer reagent (for mouse primary antibodies). After ImmPRESS polymer reagent incubation, the tissue sections were incubated in ImmPACT DAB EqV working solution until desired stain intensity developed, then the tissue sections were counterstained with hematoxylin, mounted with mounting medium, and imaged with an Olympus microscope.

Supplementary Figures

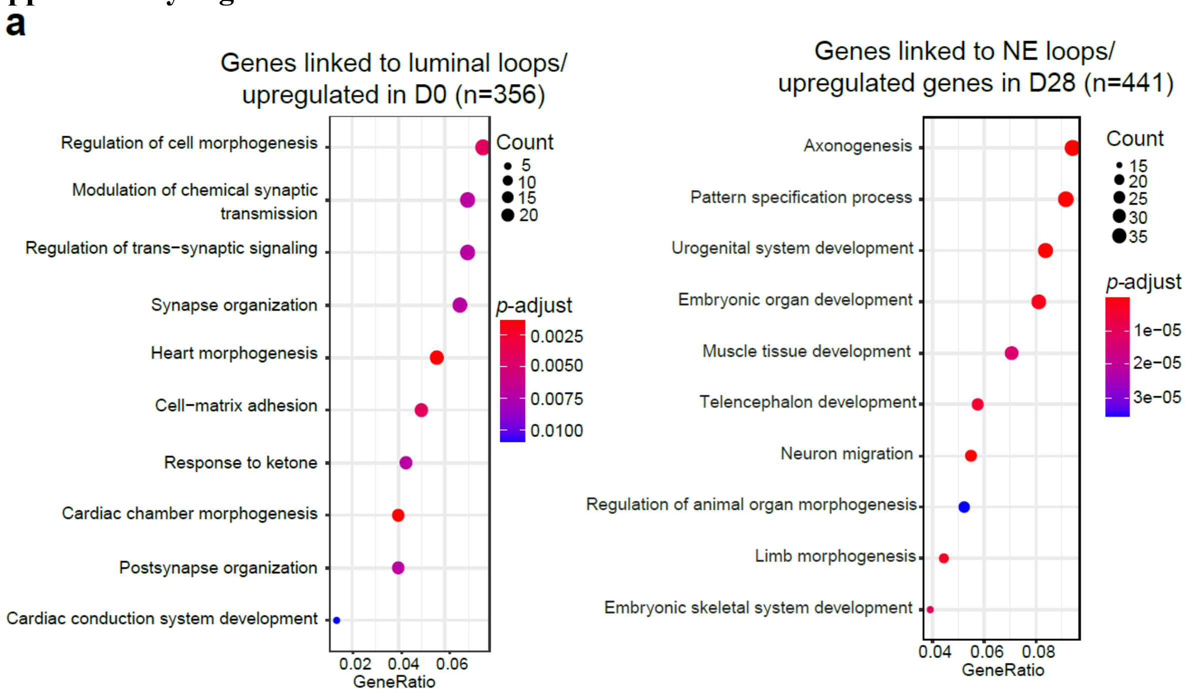

Supplementary Fig.1. GO analyses of genes linked to luminal loops and NE loops.
a. GO analyses of genes linked to luminal loops and up-regulated in D0 (**left**) and genes linked to NE loops and up-regulated in D28 (**right**).

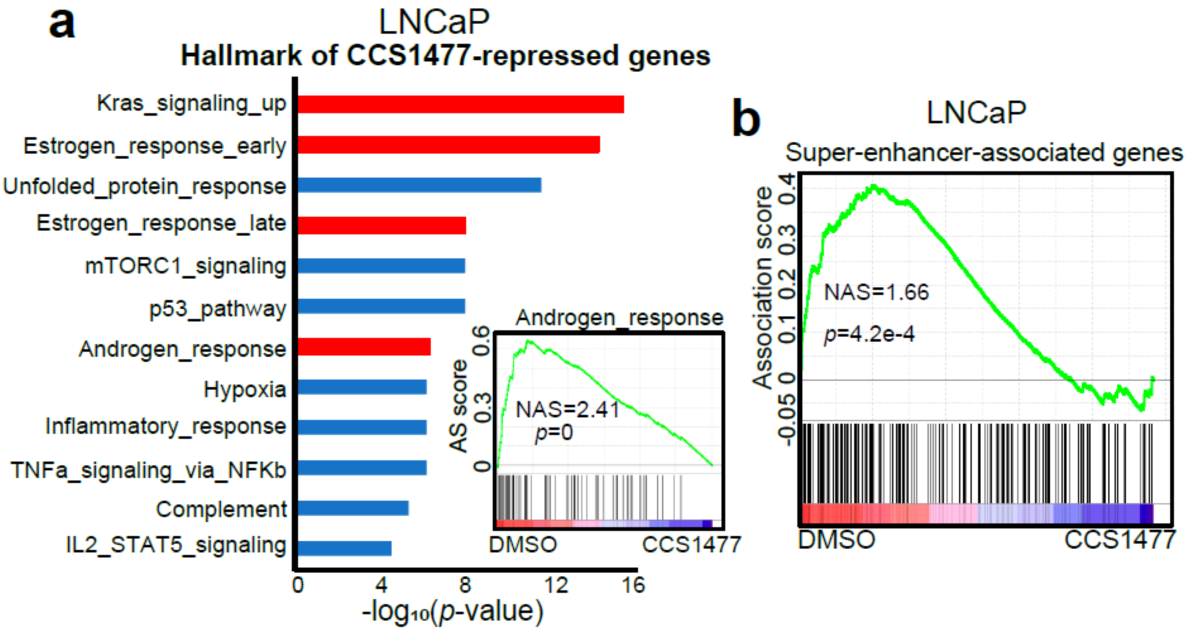

Supplementary Fig.2. CCS1477 targets lineage-specific enhancers active in a particular cell.

a. Hallmark and GSEA (inset) analyses of CCS1477-repressed genes in LNCaP cells. *P* values in Hallmark analysis were calculated by one-sided hypergeometric test. *P* value in GSEA analysis was calculated by two-sided permutation test with Benjamini-Hochberg correction.

b. GSEA showing that genes associated with SEs of LNCaP cells are strongly enriched for reduced expression in LNCaP cells treated with CCS1477, in comparison to DMSO. *P* value by two-sided permutation test with Benjamini-Hochberg correction.

Supplementary References

1 Mu, P. *et al.* SOX2 promotes lineage plasticity and antiandrogen resistance in TP53- and RB1-deficient prostate cancer. *Science* **355**, 84-88 (2017). https://doi.org:10.1126/science.aah4307

2 Bolis, M. *et al.* Dynamic prostate cancer transcriptome analysis delineates the trajectory to disease progression. *Nat Commun* **12**, 7033 (2021). https://doi.org:10.1038/s41467-021-26840-5

3 Cejas, P. *et al.* Subtype heterogeneity and epigenetic convergence in neuroendocrine prostate cancer. *Nat Commun* **12**, 5775 (2021). https://doi.org:10.1038/s41467-021-26042-z

4 Cheng, Q. *et al.* Pre-existing Castration-resistant Prostate Cancer-like Cells in Primary Prostate Cancer Promote Resistance to Hormonal Therapy. *Eur Urol* **81**, 446-455 (2022). https://doi.org:10.1016/j.eururo.2021.12.039

5 Minussi, D. C. *et al.* Breast tumours maintain a reservoir of subclonal diversity during expansion. *Nature* **592**, 302-308 (2021). https://doi.org:10.1038/s41586-021-03357-x

6 Horning, A. M. *et al.* Single-Cell RNA-seq Reveals a Subpopulation of Prostate Cancer Cells with Enhanced Cell-Cycle-Related Transcription and Attenuated Androgen Response. *Cancer Res* **78**, 853-864 (2018). https://doi.org:10.1158/0008-5472.CAN-17-1924

7 Qin, J. *et al.* The PSA(-/lo) prostate cancer cell population harbors self-renewing long-term tumor-propagating cells that resist castration. *Cell Stem Cell* **10**, 556-569 (2012). https://doi.org:10.1016/j.stem.2012.03.009

8 Gao, R. *et al.* Delineating copy number and clonal substructure in human tumors from single-cell transcriptomes. *Nat Biotechnol* **39**, 599-608 (2021). https://doi.org:10.1038/s41587-020-00795-2

9 Shiau, C. K. *et al.* High throughput single cell long-read sequencing analyses of same-cell genotypes and phenotypes in human tumors. *Nat Commun* **14**, 4124 (2023). https://doi.org:10.1038/s41467-023-39813-7

10 Baca, S. C. *et al.* Reprogramming of the FOXA1 cistrome in treatment-emergent neuroendocrine prostate cancer. *Nat Commun* **12**, 1979 (2021). https://doi.org:10.1038/s41467-021-22139-7

11 Welti, J. *et al.* Targeting the p300/CBP Axis in Lethal Prostate Cancer. *Cancer Discov* **11**, 1118-1137 (2021). https://doi.org:10.1158/2159-8290.CD-20-0751

12 Luo, J. *et al.* p300/CBP degradation is required to disable the active AR enhanceosome in prostate cancer. *bioRxiv* (2024). https://doi.org:10.1101/2024.03.29.587346

13 Loven, J. *et al.* Selective inhibition of tumor oncogenes by disruption of super-enhancers. *Cell* **153**, 320-334 (2013). https://doi.org:10.1016/j.cell.2013.03.036

14 Whyte, W. A. *et al.* Master transcription factors and mediator establish super-enhancers at key cell identity genes. *Cell* **153**, 307-319 (2013). https://doi.org:10.1016/j.cell.2013.03.035

15 Deschamps, J. & Duboule, D. Embryonic timing, axial stem cells, chromatin dynamics, and the Hox clock. *Genes Dev* **31**, 1406-1416 (2017). https://doi.org:10.1101/gad.303123.117

16 Philippidou, P. & Dasen, J. S. Hox genes: choreographers in neural development, architects of circuit organization. *Neuron* **80**, 12-34 (2013). https://doi.org:10.1016/j.neuron.2013.09.020

17 Inoue, T. *et al.* Mouse Zic5 deficiency results in neural tube defects and hypoplasia of cephalic neural crest derivatives. *Dev Biol* **270**, 146-162 (2004). https://doi.org:10.1016/j.ydbio.2004.02.017

18 Ku, S. Y. *et al.* Rb1 and Trp53 cooperate to suppress prostate cancer lineage plasticity, metastasis, and antiandrogen resistance. *Science* **355**, 78-83 (2017). https://doi.org:10.1126/science.aah4199

19 Denny, S. K. *et al.* Nfib Promotes Metastasis through a Widespread Increase in Chromatin Accessibility. *Cell* **166**, 328-342 (2016). https://doi.org:10.1016/j.cell.2016.05.052

20 Dooley, A. L. *et al.* Nuclear factor I/B is an oncogene in small cell lung cancer. *Genes Dev* **25**, 1470-1475 (2011). https://doi.org:10.1101/gad.2046711
21 Lu, X. *et al.* HOXB13 suppresses de novo lipogenesis through HDAC3-mediated epigenetic reprogramming in prostate cancer. *Nat Genet* **54**, 670-683 (2022). https://doi.org:10.1038/s41588-022-01045-8
22 Xu, B. *et al.* Altered chromatin recruitment by FOXA1 mutations promotes androgen independence and prostate cancer progression. *Cell Res* **29**, 773-775 (2019). https://doi.org:10.1038/s41422-019-0204-1
23 Drost, J. *et al.* Organoid culture systems for prostate epithelial and cancer tissue. *Nat Protoc* **11**, 347-358 (2016). https://doi.org:10.1038/nprot.2016.006
24 Corces, M. R. *et al.* An improved ATAC-seq protocol reduces background and enables interrogation of frozen tissues. *Nat Methods* **14**, 959-962 (2017). https://doi.org:10.1038/nmeth.4396
25 McLean, C. Y. *et al.* GREAT improves functional interpretation of cis-regulatory regions. *Nat Biotechnol* **28**, 495-501 (2010). https://doi.org:10.1038/nbt.1630
26 Linderman, G. C. *et al.* Zero-preserving imputation of single-cell RNA-seq data. *Nat Commun* **13**, 192 (2022). https://doi.org:10.1038/s41467-021-27729-z
27 Altemose, N. *et al.* DiMeLo-seq: a long-read, single-molecule method for mapping protein-DNA interactions genome wide. *Nat Methods* **19**, 711-723 (2022). https://doi.org:10.1038/s41592-022-01475-6
