# Supplementary material for: NKX2-1 drives neuroendocrine transdifferentiation of prostate cancer via epigenetic and 3D chromatin remodeling
Source: Nat Genet. 2025 Jul 21;57(8):1966–80. doi: 10.1038/s41588-025-02265-4 (PMC12339387; doi:10.1038/s41588-025-02265-4)
Supplement: Supplementary file 2 — Reporting Summary [file 41588_2025_2265_MOESM2_ESM.pdf]

nature portfolio

Corresponding author(s): Jindan Yu

Last updated by author(s): Mar 15, 2025

Reporting Summary

Nature Portfolio wishes to improve the reproducibility of the work that we publish. This form provides structure for consistency and transparency in reporting. For further information on Nature Portfolio policies, see our Editorial Policies and the Editorial Policy Checklist.

Statistics

For all statistical analyses, confirm that the following items are present in the figure legend, table legend, main text, or Methods section.

n/a	Confirmed	
☐	☒	The exact sample size (*n*) for each experimental group/condition, given as a discrete number and unit of measurement
☐	☒	A statement on whether measurements were taken from distinct samples or whether the same sample was measured repeatedly
☐	☒	The statistical test(s) used AND whether they are one- or two-sided<br>*Only common tests should be described solely by name; describe more complex techniques in the Methods section.*
☒	☐	A description of all covariates tested
☐	☒	A description of any assumptions or corrections, such as tests of normality and adjustment for multiple comparisons
☐	☒	A full description of the statistical parameters including central tendency (e.g. means) or other basic estimates (e.g. regression coefficient) AND variation (e.g. standard deviation) or associated estimates of uncertainty (e.g. confidence intervals)
☐	☒	For null hypothesis testing, the test statistic (e.g. *F*, *t*, *r*) with confidence intervals, effect sizes, degrees of freedom and *P* value noted<br>*Give P values as exact values whenever suitable.*
☒	☐	For Bayesian analysis, information on the choice of priors and Markov chain Monte Carlo settings
☐	☒	For hierarchical and complex designs, identification of the appropriate level for tests and full reporting of outcomes
☐	☒	Estimates of effect sizes (e.g. Cohen's *d*, Pearson's *r*), indicating how they were calculated

Our web collection on statistics for biologists contains articles on many of the points above.

Software and code

Policy information about availability of computer code

Data collection	Western Blot and Quantification: Bio-Rad ChemiDocTM with Image lab6.0.1<br>QPCR:Applied Biosystems with StepOneTM software v2.3 and QuantStudio™ Design & Analysis Software (DA2.7.0)<br>NGS:HiSEQ4000 platform with Illumina RTA 2.7.7; NOVASEQ6000,S4 Reagent Kit v1.5; NOVASEQ X PLUS 10B Reagent Kit (Cat# 20085594).<br>Oxford Nanopore Long Read Sequencing: GridION X5; Ligation Sequencing Kit: SQK-LSK110; Flow cell (R9.4.1)<br>IHC and H&E image acquisition: TissueGnostics with HistoFaxs software package/Keyence BZ-X810<br>Mass spectrometry: Orbitrap Velos Pro™ system
Data analysis	The SEQUEST was used for protein identification and peptide sequencing in FOXA2 and IgG mass spectrometry<br>For ChIP-seq analysis:<br>ChIP-seq reads were aligned to the Human Reference Genome (assembly hg19) using Bowtie2/2.0.5.<br>FastQC/0.11.5 was used to check data quality.<br>ChIP-seq peak identification, overlapping, subtraction and feature annotation of enriched regions were performed using HOMER/4.8.3 (Hypergeometric Optimization of Motif EnRichment) suite. Weighted Venn diagrams were created by R package Vennerable/3.0. Heatmap views of ChIP-seq were generated by deepTools/2.0.<br>Genomic distribution of ChIP-seq binding sites was generated by the R Bioconductor package ChIPseeker/1.36.<br>For Bulk RNA-seq analysis:<br>RNA-seq reads were mapped to NCBI human genome GRCh38 using STAR/2.6.0. Raw counts of genes were calculated by STAR. Differential gene expression was analyzed by DESeq2/1.26.0 using LTR test.<br>Heatmaps were generated by ComplexHeatmap/2.2.0.<br>Gene Set Enrichment Analysis was done by GSEA/4.1.0.<br>The scatterplot was generated using GSVA/1.52.3.

For ATAC-seq analysis:
The sequenced reads were trimmed, filtered, and aligned against hg38 using Bowtie2/2.0.5.
PCR duplicates and reads mapped to the mitochondrial chromosome or repeated regions were removed by with ENCODE ATAC-Seq pipeline 1.8.0/
Peak calling was performed using MACS2, with a p-value < 0.01 as the cutoff. Reproducible peaks from two biological replicates were defined as peaks with Irreproducibility Discovery Rates (IDR) < 0.05.
Differential ATAC-seq peaks were identified by DESeq2 using the LRT test.
For single-cell Multiome analysis:
Raw sequencing data were preprocessed and aligned to hg38 using Cell Ranger ARC (2.0.2).
Low-quality cells with low UMI counts and high mitochondrial ratios were filtered out in Seurat/4.3.0
Each time point was analyzed individually at the expression and accessibility modality, then combined across time points within each modality using Seurat (4.3.0) and Signac (1.6.0) in R (4.0.3).
The RNA modality was normalized using SCTransform/0.4.1. ATAC peaks were called using MACS2/2.1.0 and quantified.
Batch correction was done using the Seurat V3 intergration using reciprocal PCA.
Monocle3 (0.2.3.0) was used to learn the trajectory graph and calculate the pseudotime. Velocyto (0.17.17) was used to generate spliced and unspliced matrices. RNA velocities were projected into the UMAP produced from earlier steps in Seurat, using Pyplot (matplotlib 3.7.0).
The expression and chromatin accessibility of AR and NE signatures were calculated using 'AddModuleScore' in Seurat.
Correction for dropouts was done using RunALRA() in SeuratWrappers (0.3.0)
For clonality analysis:
scRNA-Seq CNV was calculated using copyKat (1.1.0).
scNanoRNA-seq SNV analysis used single cell Nanopore sequencing analysis of Genotypes and Phenotypes Simultaneously (scNanoGPS) (v1.1)
For Hi-C analysis:
Hi-C data were processed into .mcool maps using the runHiC (0.8.6) pipeline, with chromap as the aligner.
A/B compartments were called using cooltools (0.5.4) eigs_cis function with GC content phasing at 100kb resolution. Loops were called using mustache (1.2.0) at 10kb resolution
APA plots were made with coolpup.py (1.0.0).
For Nanopore long read sequencing data analysis:
Base calling and alignment were processed by guppy/6.2.1_gpu + minimap2/2.26 + remora/2.0.0.
QC was performed by mosdepth/0.3.4 and NanoStat/1.6.0.
Aggregate modified base counts for 5mC and 6mA were performed by modbam2bed/0.9.5.
Bigwig file of IGV track was generated by bedGraphToBigWig from kentUtils/302.1.
A and CpG methylation profiles, intensity plots, heatmaps and single DNA molecule tracks were generated by dimelo/0.1.0 python package.
Differentially methylated regions (DMRs) were identified by R Bioconductor package DSS/2.48.0.
The code for all NGS and Nanopore long read sequencing data analyses performed in this paper are in the process of being uploaded to Github at https://github.com/JYULAB/NKX2-1_NEPC_project.

For manuscripts utilizing custom algorithms or software that are central to the research but not yet described in published literature, software must be made available to editors and reviewers. We strongly encourage code deposition in a community repository (e.g. GitHub). See the Nature Portfolio guidelines for submitting code & software for further information.

Data

Policy information about availability of data

All manuscripts must include a data availability statement. This statement should provide the following information, where applicable:

- Accession codes, unique identifiers, or web links for publicly available datasets
- A description of any restrictions on data availability
- For clinical datasets or third party data, please ensure that the statement adheres to our policy

All sequencing data (RNA-seq, ATAC-seq, ChIP–seq, Hi-C, RRMS, DiMeLo-seq and single cell sequencing) generated for the study have been deposited in the Gene Expression Omnibus (GSE239278) at https://www.ncbi.nlm.nih.gov/geo/query/acc.cgi?acc=GSE239278
The mass spectrometry proteomic data have been deposited to the ProteomeXchange Consortium via the PRIDE96 partner repository with the dataset identifier PXD061127 and PXD061080.
The published RNA-seq, ATAC-seq, and H3K27ac ChIP-seq data from LuCaP PDX, referenced in this study, are available in the GEO database under the accession numbers GSE126078, GSE156292, and GSE161948, respectively. The WGBS and RNA-seq data from human patients, referenced in this study, are available in dbGaP (phs001648 and phs000909.v.p1) and in the GEO database under the accession numbers GSE74685, GSE126078, GSE21034, GSE6919, and GSE77930. The scRNA-seq data from human patients, referenced in this study, are from SRA PRJNA699369.

Research involving human participants, their data, or biological material

Policy information about studies with human participants or human data. See also policy information about sex, gender (identity/presentation), and sexual orientation and race, ethnicity and racism.

Reporting on sex and gender	Not applicable
Reporting on race, ethnicity, or other socially relevant groupings	Not applicable
Population characteristics	Not applicable
Recruitment	Not applicable

Ethics oversight | Not applicable

Note that full information on the approval of the study protocol must also be provided in the manuscript.

Field-specific reporting

Please select the one below that is the best fit for your research. If you are not sure, read the appropriate sections before making your selection.

☒ Life sciences ☐ Behavioural & social sciences ☐ Ecological, evolutionary & environmental sciences

For a reference copy of the document with all sections, see nature.com/documents/nr-reporting-summary-flat.pdf

Life sciences study design

All studies must disclose on these points even when the disclosure is negative.

Sample size	No statistical methods were used to pre-determine sample sizes but our sample sizes are similar to those reported in previous publications (PMID: 36332622, PMID: 35468964). For each independent in vitro experiment, at least three technical replicates were used. All in vitro experiments were independently repeated at least two or three times. For in vivo experiments, the number of animals was determined based on the variability in tumor take rate and growth and is provided in the respective figure legends.<br>RNA-seq were performed in three biological replicates and ATAC-seq were performed in two biological replicates. One to two replicates were performed for ChIP-seq. There was a good correlation between replicates for the different experiments justifying the chosen sample size in generally.
Data exclusions	No data was excluded
Replication	The experiments were performed in two biological independent replicates that generally showed high correlations
Randomization	Animals were randomly assigned to treatment groups
Blinding	Tumor measurements in animals were performed blinded

Reporting for specific materials, systems and methods

We require information from authors about some types of materials, experimental systems and methods used in many studies. Here, indicate whether each material, system or method listed is relevant to your study. If you are not sure if a list item applies to your research, read the appropriate section before selecting a response.

Materials & experimental systems

n/a	Involved in the study	
☐	☒	Antibodies
☐	☒	Eukaryotic cell lines
☒	☐	Palaeontology and archaeology
☐	☒	Animals and other organisms
☒	☐	Clinical data
☒	☐	Dual use research of concern
☒	☐	Plants

Methods

n/a	Involved in the study	
☐	☒	ChIP-seq
☒	☐	Flow cytometry
☒	☐	MRI-based neuroimaging

Antibodies

Antibodies used

Rabbit polyclonal anti-AR Millipore Cat# 06-680 WB: 1:3000
Rabbit polyclonal anti-AR Santa Cruz Biotechnology Cat# sc-816 WB (1:3000) for mouse sample
Rabbit polyclonal anti-BRN2 Bethyl Laboratories Cat#A303-583A-M WB: 1:1000
Rabbit monoclonal anti-HOXB13 Cell Signaling Technology Cat#90944S WB: 1:5000
Rabbit recombinant anti-AR Abcam Cat#ab108341 IHC: 1:1000
Rabbit polyclonal anti-histone H3 Abcam Cat#ab1791 WB: 1:200000
Mouse monoclonal anti-NCAM/CD56 Santa Cruz Biotechnology Cat#sc-7326 WB: 1:1000
Mouse monoclonal anti-SYP/Synaptophysin Santa Cruz Biotechnology Cat#sc-17750 WB/IHC (1:1000 for WB, 1:100 for IHC)
Rabbit recombinant anti-FOXA2 Abcam Cat#ab108422 WB/IHC (1:5000 for WB, 1:1000 for IHC)
Rabbit recombinant anti-FOXA2 Abcam Cat#ab256493 ChIP/DiMeLo-seq (5μg for ChIP, 1:50 for DiMeLo-seq)
Mouse monoclonal anti-TTF1/NKX2-1 Santa Cruz Biotechnology Cat#sc-53136 IHC/Co-IP (1:100 for IHC, 1:250 for Co-IP)
Rabbit polyclonal anti-TTF-1/NKX2-1 Sigma-Aldrich Cat#07-601 WB/ChIP (1:1000 for WB, 5μg for ChIP)
Rabbit monoclonal anti- acetyl-histone H3 (Lys27) Cell Signaling Technology Cat#8173S WB/ChIP (1:1000 for WB, 1:250 for ChIP)
Rabbit polyclonal anti-histone H3 (mono methyl K4) Abcam Cat#ab8895 ChIP: 5μg
Rabbit polyclonal anti-p300 Bethyl Laboratories Cat# A300-358A-M WB/ChIP (1:1000 for WB, 5μg for ChIP)
Rabbit monoclonal anti- CBP Cell Signaling Technology Cat#7389S WB/ChIP/Co-IP (1:1000 for WB, 1:100 for ChIP, 1:100 for Co-IP)

Rabbit monoclonal anti- SOX2 Cell Signaling Technology Cat#14962S WB/IHC (1:1000 for WB, 1:500 for IHC)
Rabbit monoclonal anti- PTEN Cell Signaling Technology Cat#9188S WB: 1:1000
Mouse monoclonal anti- TP53 Cell Signaling Technology Cat#2524S WB: 1:1000
Mouse monoclonal anti-GAPDH Proteintech Cat#60004-1-Ig WB: 1:1000
Mouse monoclonal anti-β-Actin Santa Cruz Biotechnology Cat#sc-47778 WB: 1:1000
Rabbit monoclonal anti-HA-Tag Cell Signaling Technology Cat#3724S WB: 1:5000
Mouse monoclonal anti-HA-Tag Santa Cruz Biotechnology Cat# sc-7392 Co-IP: 1:100
Mouse monoclonal anti-FOXA2 Abnova Cat#H00003170-M12 Co-IP: 2µg/per Co-IP
Rabbit (DA1E) mAb IgG XP® Isotype Control Cell Signaling Technology Cat#66362 ChIP/DiMeLo-seq (1:100 for ChIP, 1:50 for DiMeLo-seq)
Rabbit monoclonal anti-Tri-Methyl-Histone H3 (Lys4) Cell Signaling Technology Cat#9751 ChIP: 1:200
Rabbit monoclonal anti-Tri-Methyl-Histone H3 (Lys27) Cell Signaling Technology Cat#9733 Cut&tag: 1:50
Rabbit polyconal anti-CTCF Sigma-Aldrich Cat#07-729 ChIP: 5µg
Rabbit monoclonal anti-ASCL1 Cell Signaling Technology Cat#10585T WB: 1:1000
Rabbit monoclonal anti-Cleaved PARP Cell Signaling Technology Cat#5625S WB: 1:1000
Rabbit monoclonal anti-Cleaved Caspase-3 Cell Signaling Technology Cat#9664S WB: 1:1000
Rabbit monoclonal anti-p21 Waf1/Cip1 Cell Signaling Technology Cat#2947S WB: 1:1000

Validation

All antibodies were validated by manufacturers and previously used in several publications.
FOXA2 (Cat# ab256493) used for ChIP-seq assay was validated in PMID: 36332622; NKX2-1 (Cat# 07-601) was validated in PMID: 34466783; H3K27ac and H3K4me3 antibodies used for ChIP-seq were validated in PMID: 34937944 ; CTCF (Cat#07-729) ChIP-seq antibody was validated in PMID: 26091879; H3K4me1 (Cat#ab8895) and H3K27me3 (Cat#9733) were broadly used for ChIP-seq and Cut&tag assays in literature.

Eukaryotic cell lines

Policy information about cell lines and Sex and Gender in Research

Cell line source(s)

Normal prostate epithelia cell line RWPE-1 and Prostate cancer cell lines LNCaP, C4-2B, 22Rv1, DU145, NCI-H660 and human embryonic kidney cell line HEK293T cells were obtained from American Type Culture Collection (ATCC). Enzalutamide-resistant AR+/PSA– cell line 42D was generated by Dr. Amina Zoubeidi group at University of British Columbia.

Authentication

Authenticated using short tandem repeat (STR)profiling

Mycoplasma contamination

Cell lines tested negative for Mycoplasm

Commonly misidentified lines
(See ICLAC register)

No commonly misidentified lines were used in this study

Animals and other research organisms

Policy information about studies involving animals; ARRIVE guidelines recommended for reporting animal research, and Sex and Gender in Research

Laboratory animals

Mouse, NOD SCID (Charles River Laboratories, Strain Code. 394), Male, 6-8 weeks

Wild animals

The study did not involve wild animals

Reporting on sex

Male

Field-collected samples

The study did not involve field-collected samples

Ethics oversight

Mouse handling and experimental procedures were approved by the Institutional Animal Care and Use Committee at Northwestern University in accordance with the US National Institutes of Health Guidelines for the Care and Use of Laboratory Animals and the Animal Welfare Act.

Note that full information on the approval of the study protocol must also be provided in the manuscript.

Plants

Seed stocks

Report on the source of all seed stocks or other plant material used. If applicable, state the seed stock centre and catalogue number. If plant specimens were collected from the field, describe the collection location, date and sampling procedures.

Novel plant genotypes

Describe the methods by which all novel plant genotypes were produced. This includes those generated by transgenic approaches, gene editing, chemical/radiation-based mutagenesis and hybridization. For transgenic lines, describe the transformation method, the number of independent lines analyzed and the generation upon which experiments were performed. For gene-edited lines, describe the editor used, the endogenous sequence targeted for editing, the targeting guide RNA sequence (if applicable) and how the editor was applied.

Authentication

Describe any authentication procedures for each seed stock used or novel genotype generated. Describe any experiments used to assess the effect of a mutation and, where applicable, how potential secondary effects (e.g. second site T-DNA insertions, mosiacism, off-target gene editing) were examined.

ChIP-seq

Data deposition

☒ Confirm that both raw and final processed data have been deposited in a public database such as GEO.

☒ Confirm that you have deposited or provided access to graph files (e.g. BED files) for the called peaks.

Data access links
May remain private before publication.

https://www.ncbi.nlm.nih.gov/geo/query/acc.cgi?acc=GSE239278
Reviewer access secure token: odqxmgucrvszzur

Files in database submission

GSM7662305 LNCaP, FOXA2-D0, H3K27ac ChIP
GSM7662306 LNCaP, FOXA2-D2, H3K27ac ChIP
GSM7662307 LNCaP, FOXA2-D7, sgNC, H3K27ac ChIP
GSM7662308 LNCaP, FOXA2-D7, sgNKX2-1, H3K27ac ChIP
GSM7662309 LNCaP, FOXA2-D14, sgNC, H3K27ac ChIP
GSM7662310 LNCaP, FOXA2-D14, sgNKX2-1, H3K27ac ChIP
GSM7662311 LNCaP, FOXA2-D21, sgNC, H3K27ac ChIP
GSM7662312 LNCaP, FOXA2-D21, sgNKX2-1, H3K27ac ChIP
GSM7662313 LNCaP, FOXA2-D28, sgNC, H3K27ac ChIP
GSM7662314 LNCaP, FOXA2-D28, sgNKX2-1, H3K27ac ChIP
GSM7662315 LNCaP, FOXA2-D0, H3K4me1 ChIP
GSM7662316 LNCaP, FOXA2-D2, H3K4me1 ChIP
GSM7662317 LNCaP, FOXA2-D7, sgNC, H3K4me1 ChIP
GSM7662318 LNCaP, FOXA2-D7, sgNKX2-1, H3K4me1 ChIP
GSM7662319 LNCaP, FOXA2-D14, sgNC, H3K4me1 ChIP
GSM7662320 LNCaP, FOXA2-D14, sgNKX2-1, H3K4me1 ChIP
GSM7662321 LNCaP, FOXA2-D21, sgNC, H3K4me1 ChIP
GSM7662322 LNCaP, FOXA2-D21, sgNKX2-1, H3K4me1 ChIP
GSM7662323 LNCaP, FOXA2-D28, sgNC, H3K4me1 ChIP
GSM7662324 LNCaP, FOXA2-D28, sgNKX2-1, H3K4me1 ChIP
GSM7662325 LNCaP, FOXA2-D0, FOXA2 ChIP
GSM7662326 LNCaP, FOXA2-D2, FOXA2 ChIP
GSM7662327 LNCaP, FOXA2-D7, sgNC, FOXA2 ChIP
GSM7662328 LNCaP, FOXA2-D7, sgNKX2-1, FOXA2 ChIP
GSM7662329 LNCaP, FOXA2-D14, sgNC, FOXA2 ChIP
GSM7662330 LNCaP, FOXA2-D14, sgNKX2-1,FOXA2 ChIP
GSM7662331 LNCaP, FOXA2-D21, sgNC, FOXA2 ChIP
GSM7662332 LNCaP, FOXA2-D21, sgNKX2-1,FOXA2 ChIP
GSM7662333 LNCaP, FOXA2-D28, sgNC, FOXA2 ChIP
GSM7662334 LNCaP, FOXA2-D28, sgNKX2-1, FOXA2 ChIP
GSM7662335 LNCaP, FOXA2-D7, sgNC, NKX2-1 ChIP
GSM7662336 LNCaP, FOXA2-D7, sgNKX2-1, NKX2-1 ChIP
GSM7662337 LNCaP, FOXA2-D14, sgNC, NKX2-1 ChIP
GSM7662338 LNCaP, FOXA2-D14, sgNKX2-1,NKX2-1 ChIP
GSM7662339 LNCaP, FOXA2-D21, sgNC, NKX2-1 ChIP
GSM7662340 LNCaP, FOXA2-D21, sgNKX2-1, NKX2-1 ChIP
GSM7662341 LNCaP, FOXA2-D28, sgNC, NKX2-1 ChIP
GSM7662342 LNCaP, FOXA2-D28, sgNKX2-1, NKX2-1 ChIP
GSM7662343 LNCaP, FOXA2-D2, CTCF ChIP
GSM7662344 LNCaP, FOXA2-D14, CTCF ChIP
GSM7662345 LNCaP, FOXA2-D21, CTCF ChIP
GSM7662346 LNCaP, FOXA2-D28, CTCF ChIP
GSM7662347 LuCaP35CR, H3K27ac ChIP
GSM7662348 LuCaP147CR, H3K27ac ChIP
GSM7662349 LuCaP93, H3K27ac ChIP
GSM7662350 LuCaP145.2, H3K27ac ChIP

GSM7662351 NCI-H660, FOXA2 ChIP 1#
GSM7662352 NCI-H660, NKX2-1 ChIP 1#
GSM7662353 LNCaP, FOXA2-D2, H3K27me3 Cut&tag
GSM7662354 LNCaP, FOXA2-D14, H3K27me3 Cut&tag
GSM7662355 LNCaP, FOXA2-D21, H3K27me3 Cut&tag
GSM7662356 LNCaP, FOXA2-D28, H3K27me3 Cut&tag
GSM7662357 LNCaP, FOXA2-D14, FOXA2 ChIP
GSM7662358 LNCaP, FOXA2+NKX2-1-D14, FOXA2 ChIP
GSM7662359 LuNE, sgNC, p300 ChIP
GSM7662360 LuNE, sgNKX2-1, p300 ChIP
GSM7662361 LuNE, sgFOXA2, p300 ChIP
GSM7662362 NCI-H660, FOXA2 ChIP 2#
GSM7662363 NCI-H660, NKX2-1 ChIP 2#
GSM7662364 LuCaP145.2, FOXA2 ChIP
GSM7662365 LuCaP145.2, NKX2-1 ChIP
GSM7662366 LuNE, DMSO, H3K27ac ChIP 1#
GSM7662367 LuNE, CCS1477, H3K27ac ChIP 1#
GSM7662368 LuNE, shCtrl, H3K27ac ChIP 1#
GSM7662369 LuNE, shp300, H3K27ac ChIP 1#
GSM7662370 LuNE, shCBP, H3K27ac ChIP 1#
GSM7662371 LuNE, DMSO, H3K27ac ChIP 2#
GSM7662372 LuNE, CCS1477, H3K27ac ChIP 2#
GSM7662373 LuNE, shCtrl, H3K27ac ChIP 2#
GSM7662374 LuNE, shp300, H3K27ac ChIP 2#
GSM7662375 LuNE, shCBP, H3K27ac ChIP 2#
GSM7662376 NCI-H660, H3K4me1 ChIP 1#
GSM7662377 NCI-H660, H3K4me1 ChIP 2#
GSM7662378 NCI-H660, H3K4me3 ChIP
GSM7662379 LuCaP145.2, H3K4me1 ChIP 1#
GSM7662380 LuCaP145.2, H3K4me1 ChIP 2#
GSM7662381 NCI-H660, H3K27ac ChIP
GSM7662382 LuNE, sgNC, H3K27ac ChIP 1#
GSM7662383 LuNE, sgNKX2-1, H3K27ac ChIP 1#
GSM7662384 LuNE, sgFOXA2, H3K27ac ChIP 1#
GSM7662385 LuNE, sgNC, H3K27ac ChIP 2#
GSM7662386 LuNE, sgNKX2-1, H3K27ac ChIP 2#
GSM7662387 LuNE, sgFOXA2, H3K27ac ChIP 2#
GSM7662388 LuNE, FOXA2 ChIP 1#
GSM7662389 LuNE, FOXA2 ChIP 2#
GSM8061324 LuCaP145.1, H3K27ac ChIP
GSM8061325 LuCaP70CR, H3K27ac ChIP
GSM8061326 LuCaP77CR, H3K27ac ChIP
GSM8061327 LuCaP35CR, H3K27me3 Cut&Tag
GSM8061328 LuCaP93, H3K27me3 Cut&Tag
GSM8061329 LuCaP145.2, H3K27me3 Cut&Tag
GSM8061330 LuCaP147, H3K27me3 Cut&Tag
GSM8061331 LuCaP70CR, H3K27me3 Cut&Tag
GSM8061332 LuCaP145.1, H3K27me3 Cut&Tag
GSM8061333 NCI-H660, H3K27me3 Cut&Tag
GSM8061334 LuCaP77CR, H3K27me3 Cut&Tag
GSM8061335 LuCaP93, NKX2-1 ChIP
GSM8061336 LuCaP145.1, H3K4me1 ChIP
GSM8061337 LuCaP145.1, FOXA2 ChIP
GSM8061338 LuCaP145.1, NKX2-1 ChIP
GSM8373006 LuCaP93, H3K4me1 ChIP

Genome browser session
(e.g. UCSC)

FOXA2, H3K4me1,H3K27ac and NKX2-1 time-course ChIP-seq tracks:
https://genome.ucsc.edu/cgi-bin/hgTracks?db=hg19&lastVirtModeType=default&lastVirtModeExtraState=&virtModeType=default&virtMode=0&nonVirtPosition=&position=chr14%3A36970296%2D37004738&hgsid=1892428022_N0ACZGZ02xDNOXfQyjpoKwAA5Zmi

Methodology

Replicates

One or two replicates were performed for ChIP-seq experiments, and the data were pooled for analysis.

Sequencing depth

M893 LNCaP, FOXA2-D0, H3K27ac ChIP 50bp single
M894 LNCaP, FOXA2-D2, H3K27ac ChIP 50bp single
M895 LNCaP, FOXA2-D7, sgNC, H3K27ac ChIP 50bp single
M896 LNCaP, FOXA2-D7, sgNKX2-1, H3K27ac ChIP 50bp single
M897 LNCaP, FOXA2-D14, sgNC, H3K27ac ChIP 50bp single
M898 LNCaP, FOXA2-D14, sgNKX2-1, H3K27ac ChIP 50bp single
M899 LNCaP, FOXA2-D21, sgNC, H3K27ac ChIP 50bp single
M900 LNCaP, FOXA2-D21, sgNKX2-1, H3K27ac ChIP 50bp single
M901 LNCaP, FOXA2-D28, sgNC, H3K27ac ChIP 50bp single
M902 LNCaP, FOXA2-D28, sgNKX2-1, H3K27ac ChIP 50bp single

M903 LNCaP, FOXA2-D0, H3K4me1 ChIP 50bp single
M904 LNCaP, FOXA2-D2, H3K4me1 ChIP 50bp single
M905 LNCaP, FOXA2-D7, sgNC, H3K4me1 ChIP 50bp single
M906 LNCaP, FOXA2-D7, sgNKX2-1, H3K4me1 ChIP 50bp single
M1390 LNCaP,DMSO,H3K27ac ChIP 150bp paired
M1392 LNCaP,CCS1477,H3K27ac ChIP 150bp paired

M907 LNCaP, FOXA2-D14, sgNC, H3K4me1 ChIP 50bp single
M908 LNCaP, FOXA2-D14, sgNKX2-1, H3K4me1 ChIP 50bp single
M909 LNCaP, FOXA2-D21, sgNC, H3K4me1 ChIP 50bp single
M910 LNCaP, FOXA2-D21, sgNKX2-1, H3K4me1 ChIP 50bp single
M911 LNCaP, FOXA2-D28, sgNC, H3K4me1 ChIP 50bp single
M912 LNCaP, FOXA2-D28, sgNKX2-1, H3K4me1 ChIP 50bp single
M913 LNCaP, FOXA2-D0, FOXA2 ChIP 50bp single
M914 LNCaP, FOXA2-D2, FOXA2 ChIP 50bp single
M915 LNCaP, FOXA2-D7, sgNC, FOXA2 ChIP 50bp single
M916 LNCaP, FOXA2-D7, sgNKX2-1, FOXA2 ChIP 50bp single
M917 LNCaP, FOXA2-D14, sgNC, FOXA2 ChIP 50bp single
M918 LNCaP, FOXA2-D14, sgNKX2-1,FOXA2 ChIP 50bp single
M919 LNCaP, FOXA2-D21, sgNC, FOXA2 ChIP 50bp single
M920 LNCaP, FOXA2-D21, sgNKX2-1,FOXA2 ChIP 50bp single
M921 LNCaP, FOXA2-D28, sgNC, FOXA2 ChIP 50bp single
M922 LNCaP, FOXA2-D28, sgNKX2-1, FOXA2 ChIP 50bp single
M923 LNCaP, FOXA2-D7, sgNC, NKX2-1 ChIP 50bp single
M924 LNCaP, FOXA2-D7, sgNKX2-1, NKX2-1 ChIP 50bp single
M925 LNCaP, FOXA2-D14, sgNC, NKX2-1 ChIP 50bp single
M926 LNCaP, FOXA2-D14, sgNKX2-1,NKX2-1 ChIP 50bp single
M927 LNCaP, FOXA2-D21, sgNC, NKX2-1 ChIP 50bp single
M928 LNCaP, FOXA2-D21, sgNKX2-1, NKX2-1 ChIP 50bp single
M929 LNCaP, FOXA2-D28, sgNC, NKX2-1 ChIP 50bp single
M930 LNCaP, FOXA2-D28, sgNKX2-1, NKX2-1 ChIP 50bp single
M1015 LNCaP, FOXA2-D2, CTCF ChIP 50bp paired-end
M1016 LNCaP, FOXA2-D14, CTCF ChIP 50bp paired-end
M1017 LNCaP, FOXA2-D21, CTCF ChIP 50bp paired-end
M1018 LNCaP, FOXA2-D28, CTCF ChIP 50bp paired-end
M994 LuCaP35CR, H3K27ac ChIP 50bp paired-end
M998 LuCaP147CR, H3K27ac ChIP 50bp paired-end
M1026 LuCaP93, H3K27ac ChIP 50bp paired-end
M1027 LuCaP145.2, H3K27ac ChIP 50bp paired-end
M1043 NCI-H660, FOXA2 ChIP 1# 50bp paired-end
M1044 NCI-H660, NKX2-1 ChIP 1# 50bp paired-end
M1047 LNCaP, FOXA2-D2, H3K27me3 Cut&tag 50bp paired-end
M1048 LNCaP, FOXA2-D14, H3K27me3 Cut&tag 50bp paired-end
M1049 LNCaP, FOXA2-D21, H3K27me3 Cut&tag 50bp paired-end
M1050 LNCaP, FOXA2-D28, H3K27me3 Cut&tag 50bp paired-end
M1205 LNCaP, FOXA2-D14, FOXA2 ChIP 50bp single
M1235 LNCaP, FOXA2+NKX2-1-D14, FOXA2 ChIP 50bp paired-end
M1241 LuNE, sgNC, P300 ChIP 50bp paired-end
M1242 LuNE, sgNKX2-1, P300 ChIP 50bp paired-end
M1243 LuNE, sgFOXA2, P300 ChIP 50bp paired-end
M1135 NCI-H660, FOXA2 ChIP 2# 50bp paired-end
M1136 NCI-H660, NKX2-1 ChIP 2# 50bp paired-end
M1380 LuCaP145.2, FOXA2 ChIP 50bp paired-end
M1152 LuCaP145.2, NKX2-1 ChIP 50bp paired-end
M1331 LuNE, DMSO, H3K27ac ChIP 1# 50bp single
M1332 LuNE, CCS1477, H3K27ac ChIP 1# 50bp single
M1335 LuNE, shCtrl, H3K27ac ChIP 1# 50bp single
M1336 LuNE, shP300, H3K27ac ChIP 1# 50bp single
M1337 LuNE, shCBP, H3K27ac ChIP 1# 50bp single
M1323 LuNE, DMSO, H3K27ac ChIP 2# 50bp paired-end
M1324 LuNE, CCS1477, H3K27ac ChIP 2# 50bp paired-end
M1327 LuNE, shCtrl, H3K27ac ChIP 2# 50bp paired-end
M1328 LuNE, shP300, H3K27ac ChIP 2# 50bp paired-end
M1329 LuNE, shCBP, H3K27ac ChIP 2# 50bp paired-end
M1377 NCI-H660, H3K4me1 ChIP 1# 50bp paired-end
M1378 NCI-H660, H3K4me1 ChIP 2# 50bp paired-end
M1379 NCI-H660, H3K4me3 ChIP 50bp paired-end
M1382 LuCaP145.2, H3K4me1 ChIP 1# 50bp paired-end
M1383 LuCaP145.2, H3K4me1 ChIP 2# 50bp paired-end
M1131 NCI-H660, H3K27ac ChIP 50bp paired-end
M1407 LuNE, sgNC, H3K27ac ChIP 1# 150bp paired-end
M1408 LuNE, sgNKX2-1, H3K27ac ChIP 1# 150bp paired-end
M1409 LuNE, sgFOXA2, H3K27ac ChIP 1# 150bp paired-end
M1411 LuNE, sgNC, H3K27ac ChIP 2# 150bp paired-end
M1412 LuNE, sgNKX2-1, H3K27ac ChIP 2# 150bp paired-end
M1413 LuNE, sgFOXA2, H3K27ac ChIP 2# 150bp paired-end

M1103 LuNE, FOXA2 ChIP 1# 50bp paired-end
M1108 LuNE, FOXA2 ChIP 2# 50bp paired-end
M1507 LuCaP145.1, H3K27ac ChIP 150bp paired-end
M995 LuCaP70CR, H3K27ac ChIP 50bp paired-end
M996 LuCaP77CR, H3K27ac ChIP 50bp paired-end
M1466 LuCaP35CR, H3K27me3 Cut&Tag 150bp paired-end
M1467 LuCaP93, H3K27me3 Cut&Tag 150bp paired-end
M1468 LuCaP145.2, H3K27me3 Cut&Tag 150bp paired-end
M1469 LuCaP147, H3K27me3 Cut&Tag 150bp paired-end
M1496 LuCaP70CR, H3K27me3 Cut&Tag 150bp paired-end
M1497 LuCaP145.1, H3K27me3 Cut&Tag 150bp paired-end
M1498 NCI-H660, H3K27me3 Cut&Tag 150bp paired-end
M1529 LuCaP77CR, H3K27me3 Cut&Tag 150bp paired-end
M1505 LuCaP93, NKX2-1 ChIP 150bp paired-end
M1506 LuCaP145.1, H3K4me1 ChIP 150bp paired-end
M1508 LuCaP145.1, FOXA2 ChIP 150bp paired-end
M1509 LuCaP145.1, NKX2-1 ChIP 150bp paired-end
M1457 LuCaP93, H3K4me1 ChIP 150bp paired-end

Antibodies

Rabbit recombinant anti-FOXA2 Abcam Cat#ab256493 ChIP/DiMeLo-seq
Rabbit polyclonal anti-TTF-1/NKX2-1 Sigma-Aldrich Cat#07-601 ChIP
Rabbit monoclonal anti- acetyl-histone H3 (Lys27) Cell Signaling Technology Cat#8173S ChIP
Rabbit polyclonal anti-histone H3 (mono methyl K4) Abcam Cat#ab8895 ChIP
Rabbit polyclonal anti-p300 Bethyl Laboratories Cat# A300-358A-M ChIP
Rabbit (DA1E) mAb IgG XP® Isotype Control Cell Signaling Technology Cat#66362 ChIP/DiMeLo-seq
Rabbit monoclonal anti-Tri-Methyl-Histone H3 (Lys4) Cell Signaling Technology Cat#9751 ChIP
Rabbit monoclonal anti-Tri-Methyl-Histone H3 (Lys27) Cell Signaling Technology Cat#9733 Cut&Tag
Rabbit polyconal anti-CTCF Sigma-Aldrich Cat#07-729 ChIP

Peak calling parameters

Bowtie2, HOMER and MACS2, all used default parameters.

Data quality

Raw sequencing quality was assessed with FastQC/0.11.5, cross-correlation enrichment metrics were checked by deepTools/2.0 plotCorrelation tool.

Software

FastQC/0.11.5
samtools/1.6
Bowtie2/2.0.5
HOMER/4.8.3
MACS/2.0
samtools/1.6
Vennerable/3.0
deepTools/2.0
