# Supplementary figures and images for: NKX2-1 drives neuroendocrine transdifferentiation of prostate cancer via epigenetic and 3D chromatin remodeling
Source: Nat Genet. 2025 Jul 21;57(8):1966–80. doi: 10.1038/s41588-025-02265-4 (PMC12339387; doi:10.1038/s41588-025-02265-4)

Fig.2b

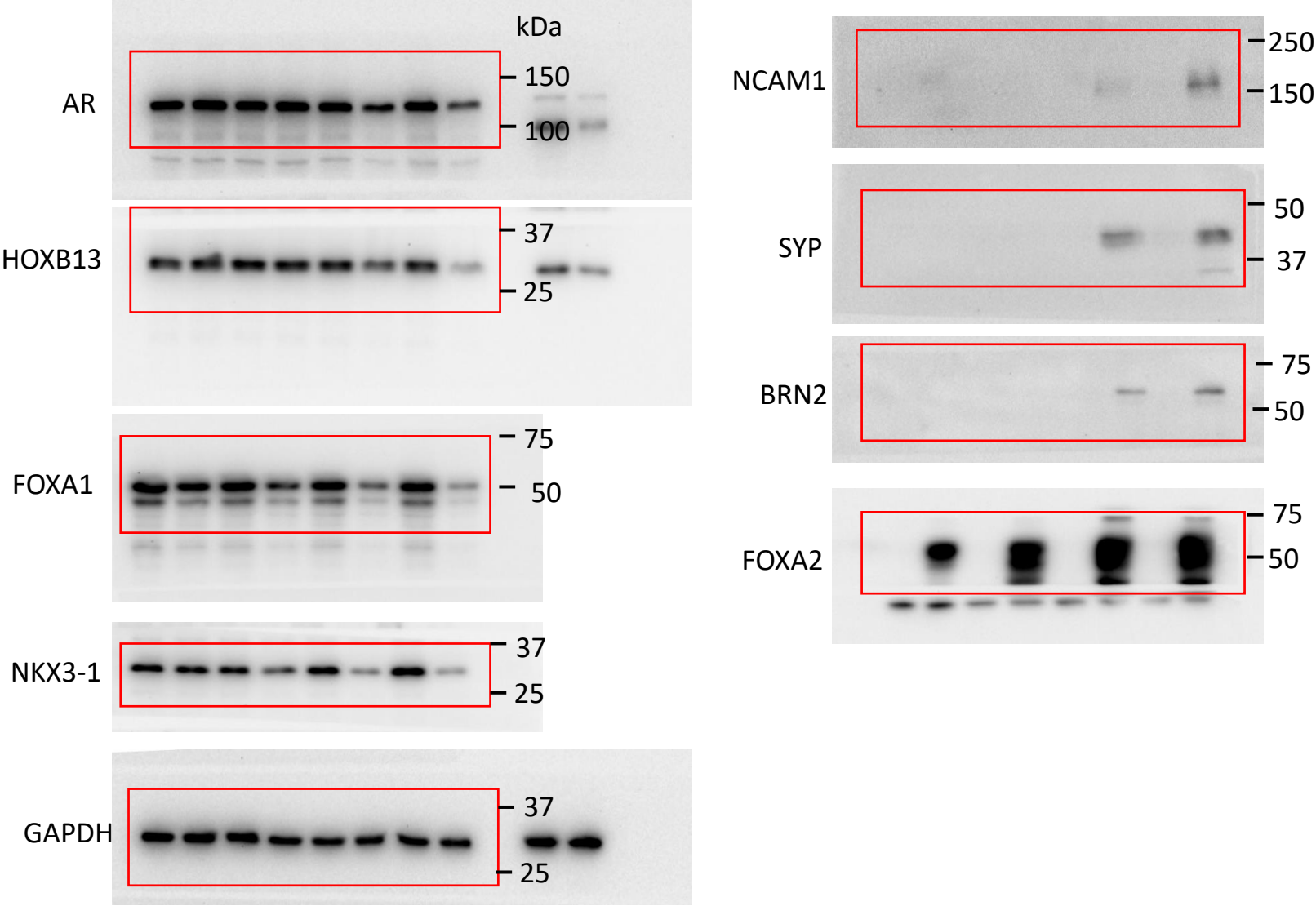

Fig.4b

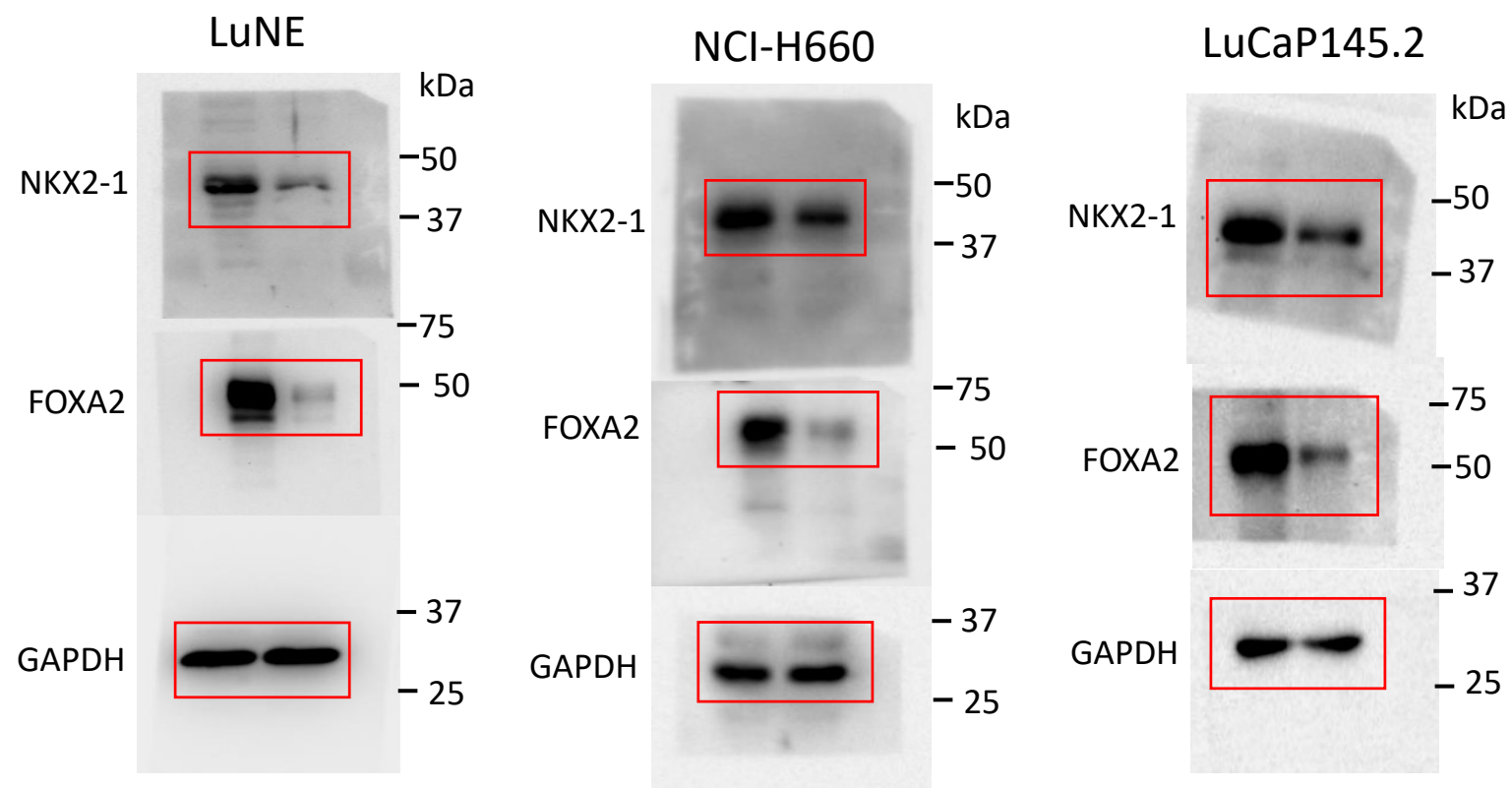

Fig.4c

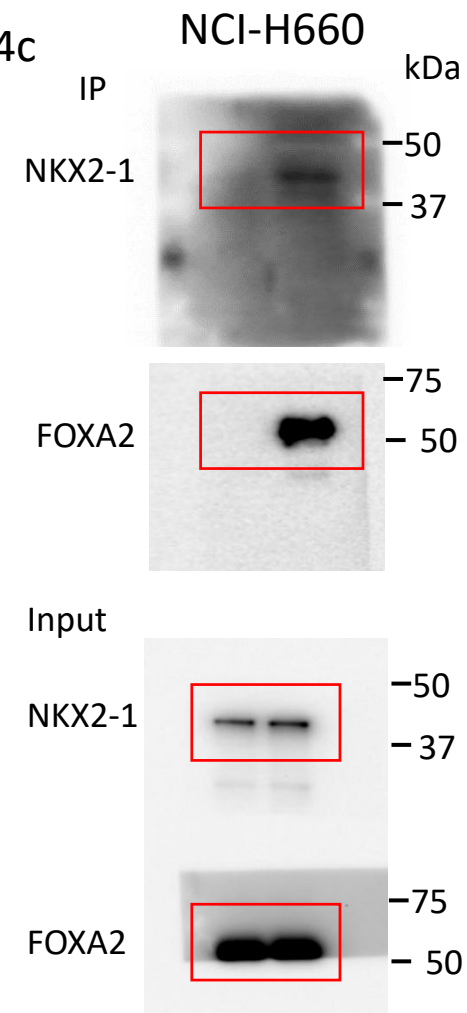

Fig.4g

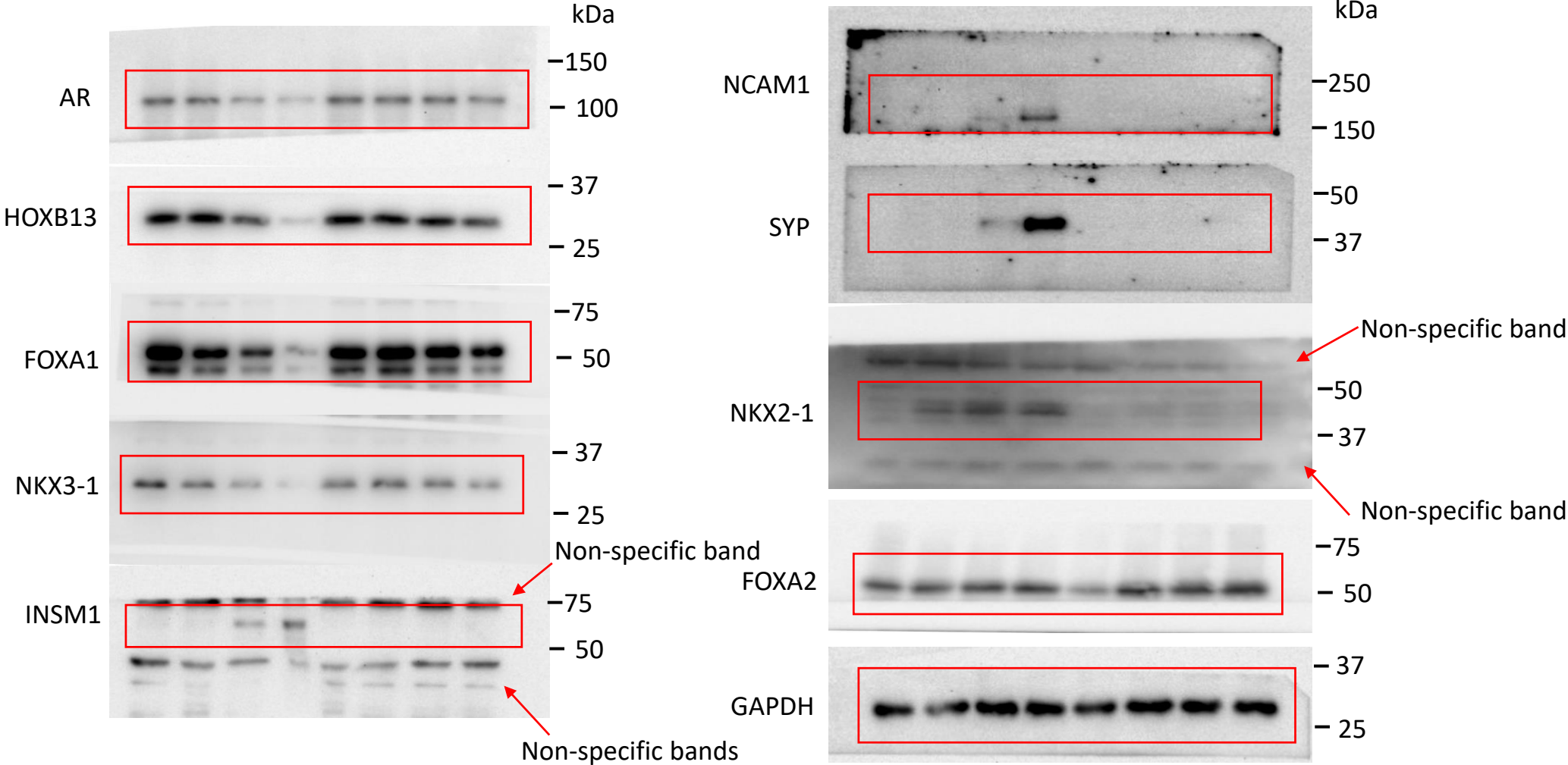

Fig.5d

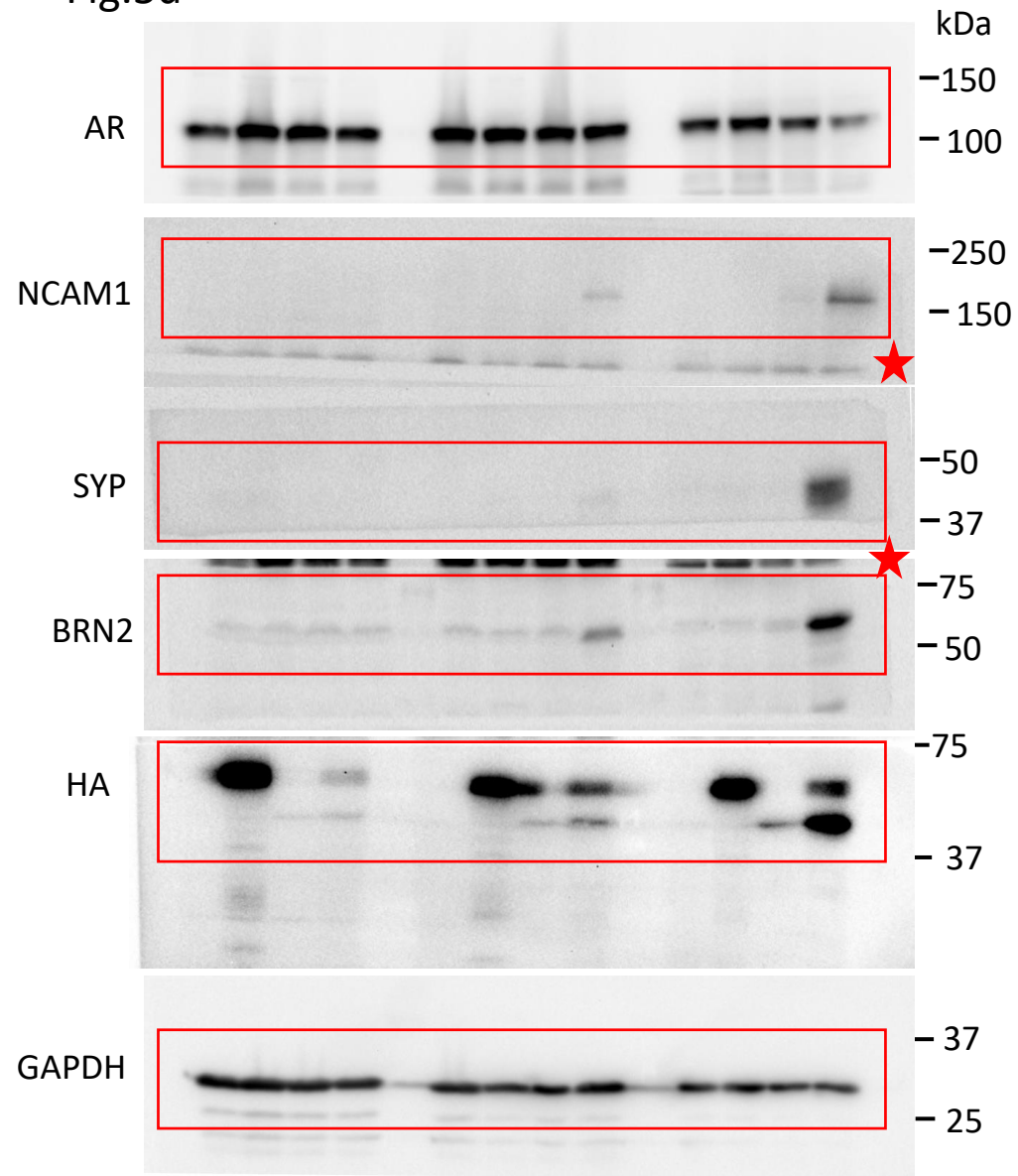

★ Non-specific band

Fig.5f

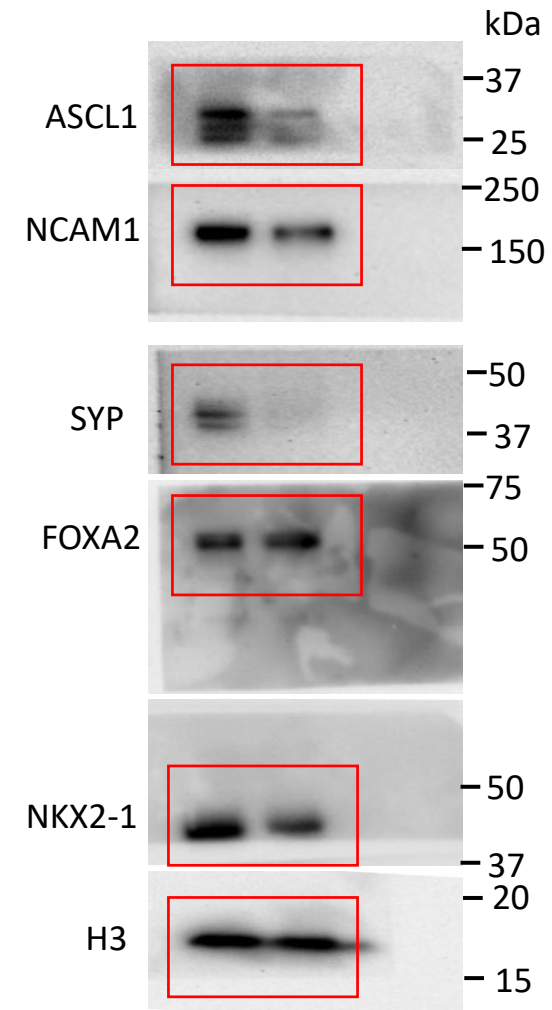

Fig.5g

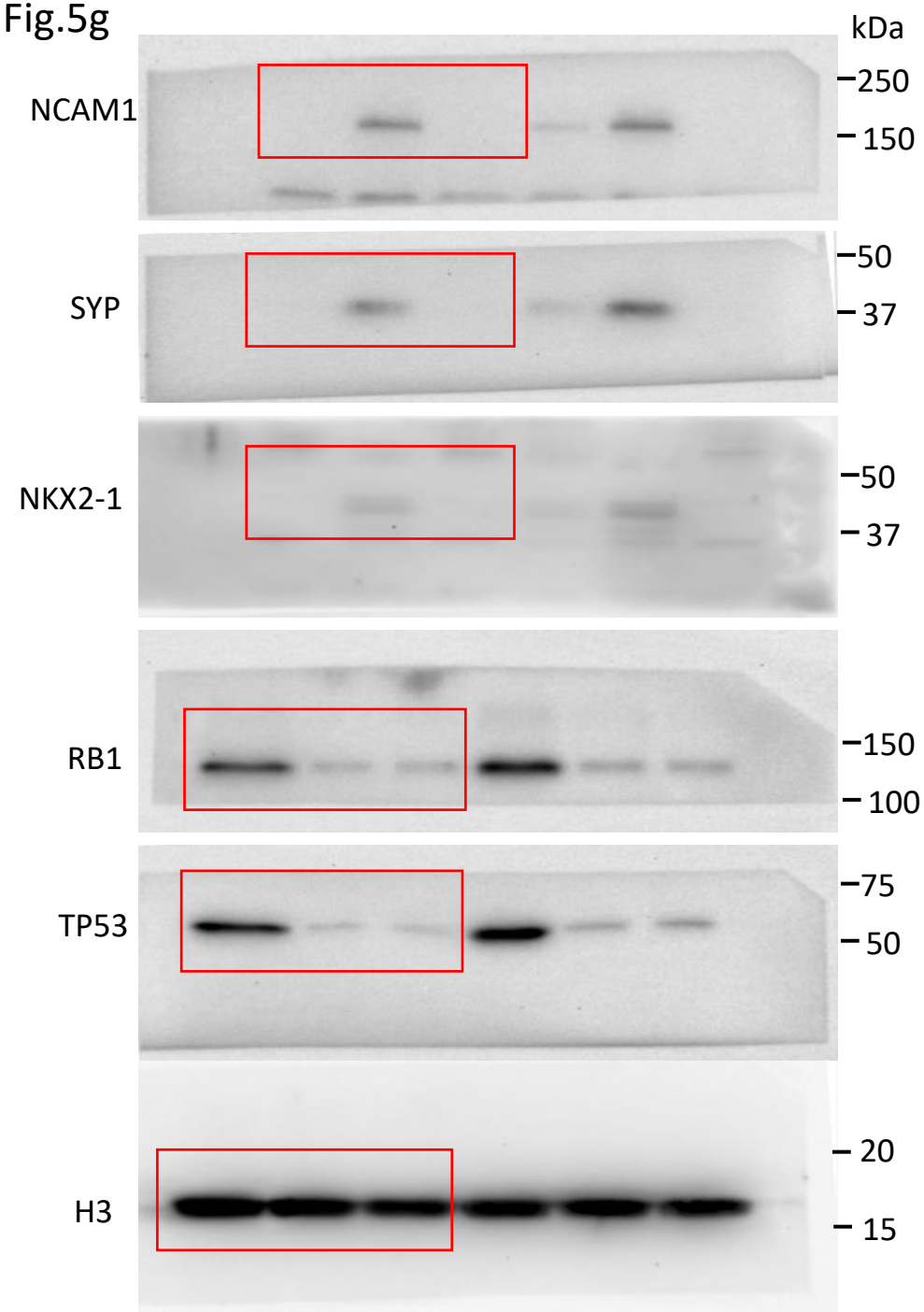

Fig.5h

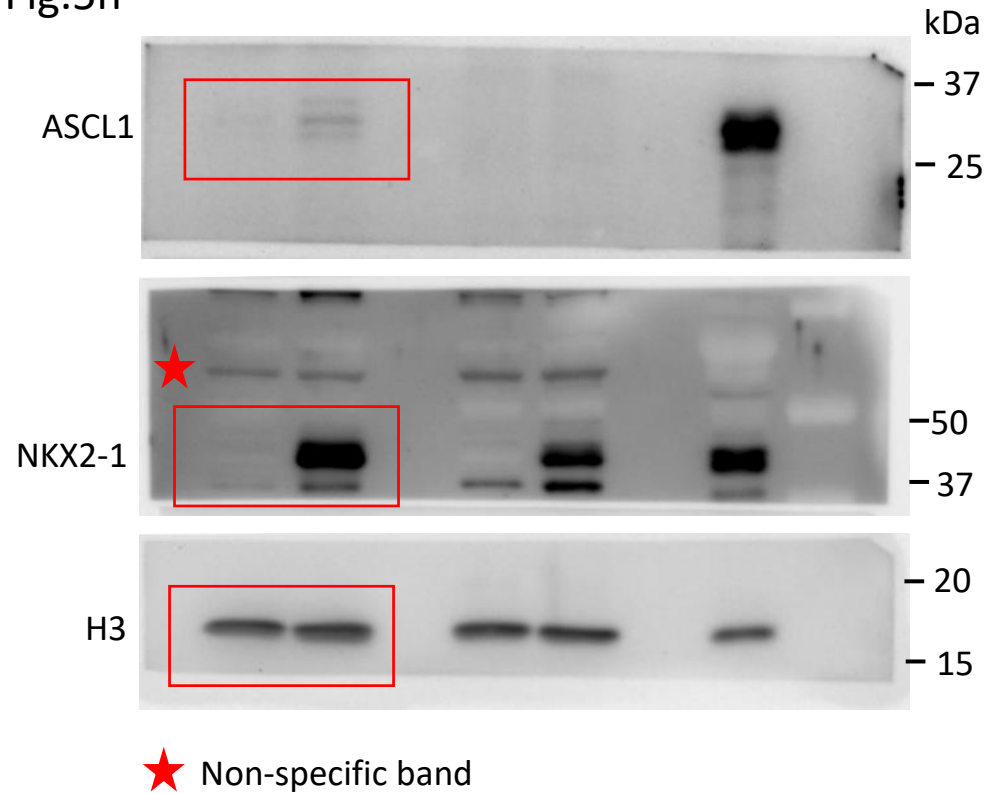

Fig.7b

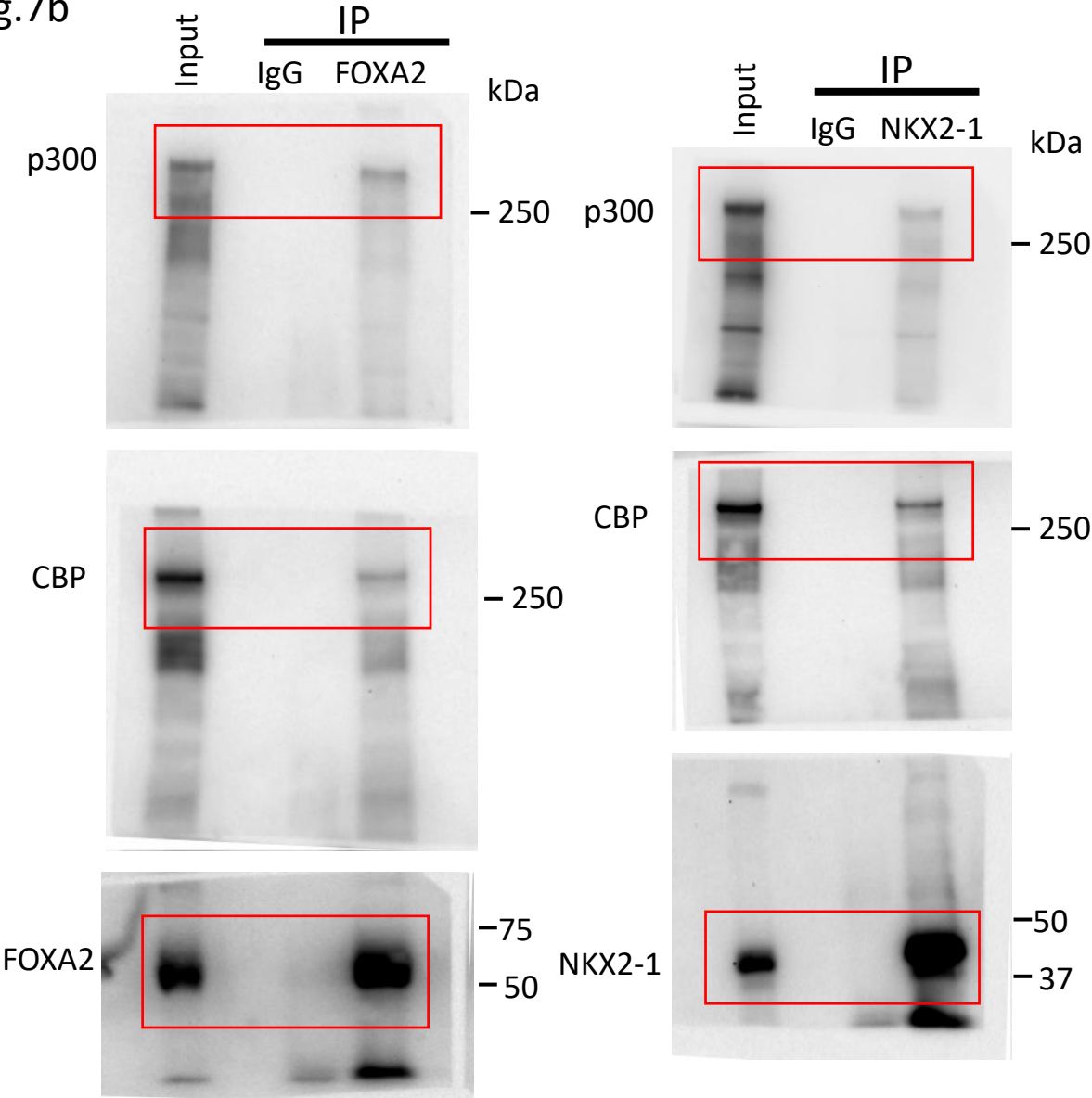

Fig.7c

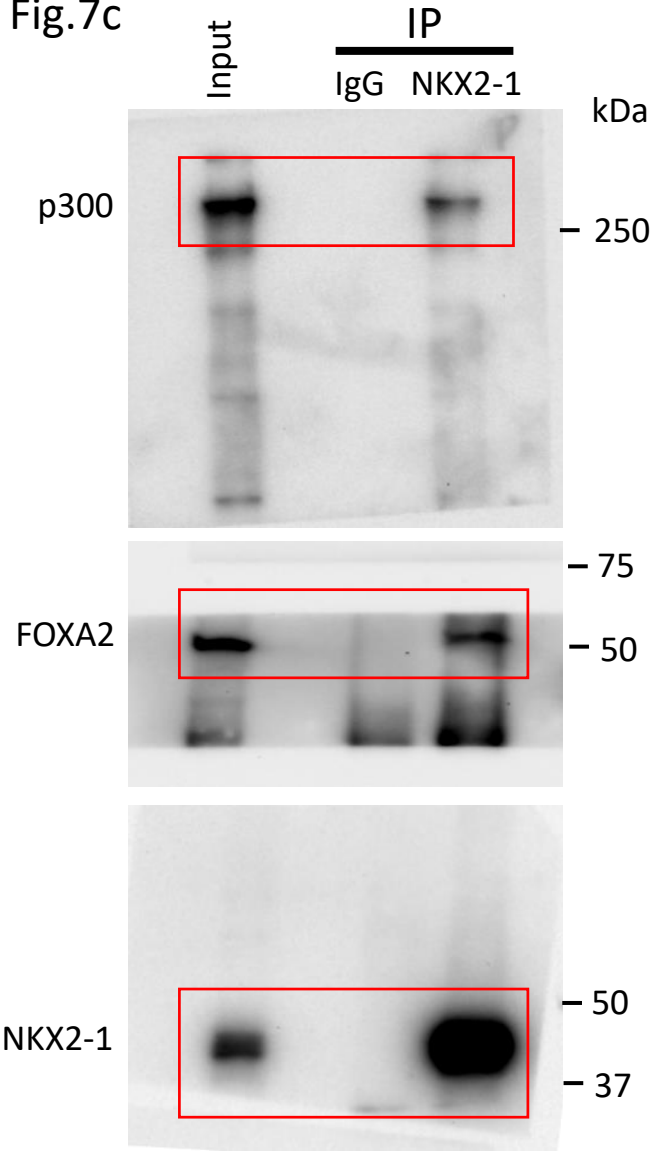

Supplement: Supplementary file 9 — Unprocessed WBs for Figs. 2b, 4b,c,g, 5d,f–h and 7b,c. [file 41588_2025_2265_MOESM9_ESM.pdf]
