# Supplementary material for: NKX2-1 drives neuroendocrine transdifferentiation of prostate cancer via epigenetic and 3D chromatin remodeling
Source: Nat Genet. 2025 Jul 21;57(8):1966–80. doi: 10.1038/s41588-025-02265-4 (PMC12339387; doi:10.1038/s41588-025-02265-4)
Supplement: Supplementary file 10 — Unprocessed WBs for Extended Data Figs. 2b,c, 7d,g, 9g and 10d,j. [file 41588_2025_2265_MOESM10_ESM.pdf]

## Extended Data Fig.2b

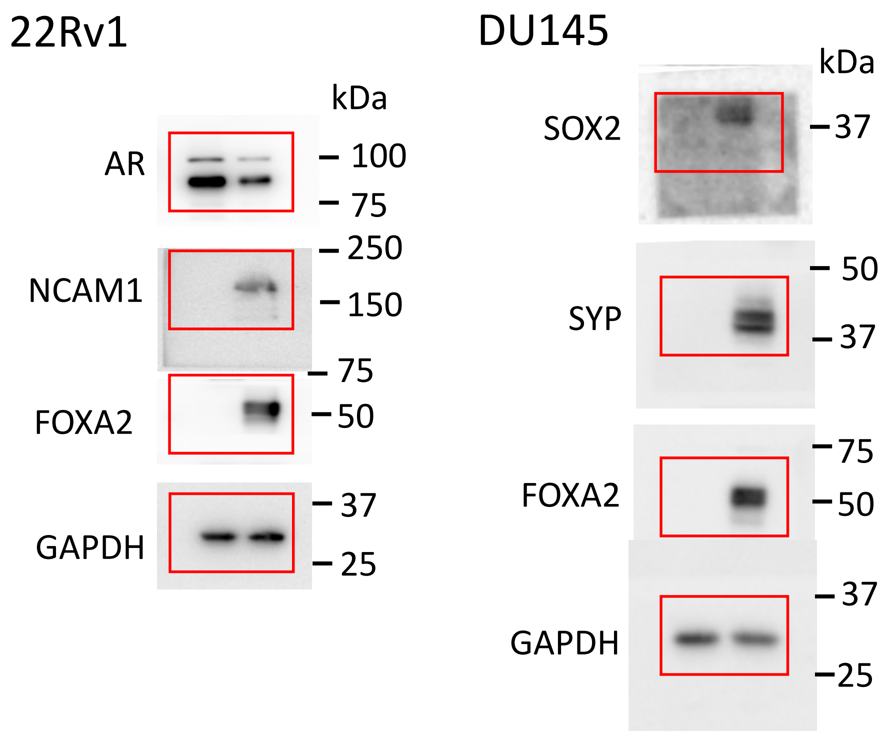


## Extended Data Fig.2c

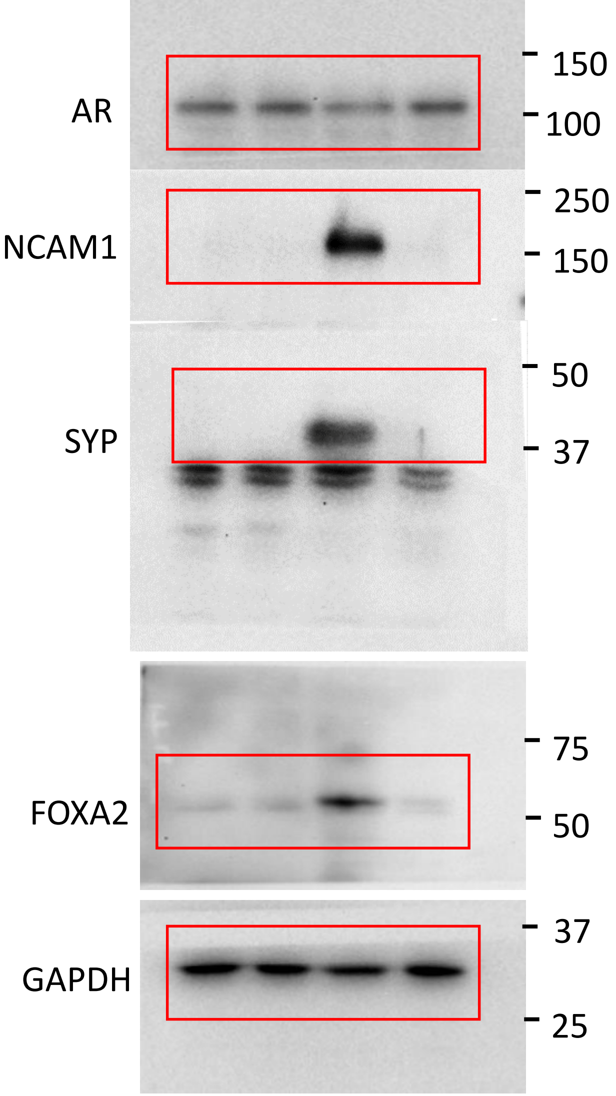

Extended Data Fig.7d

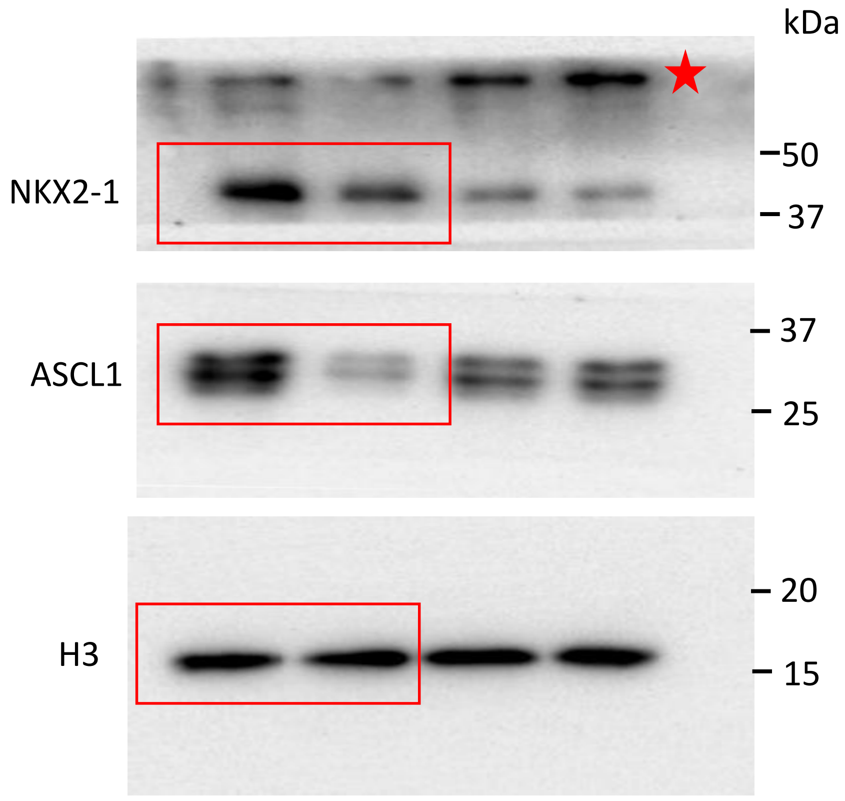


★ Non-specific band

Extended Data Fig.7g

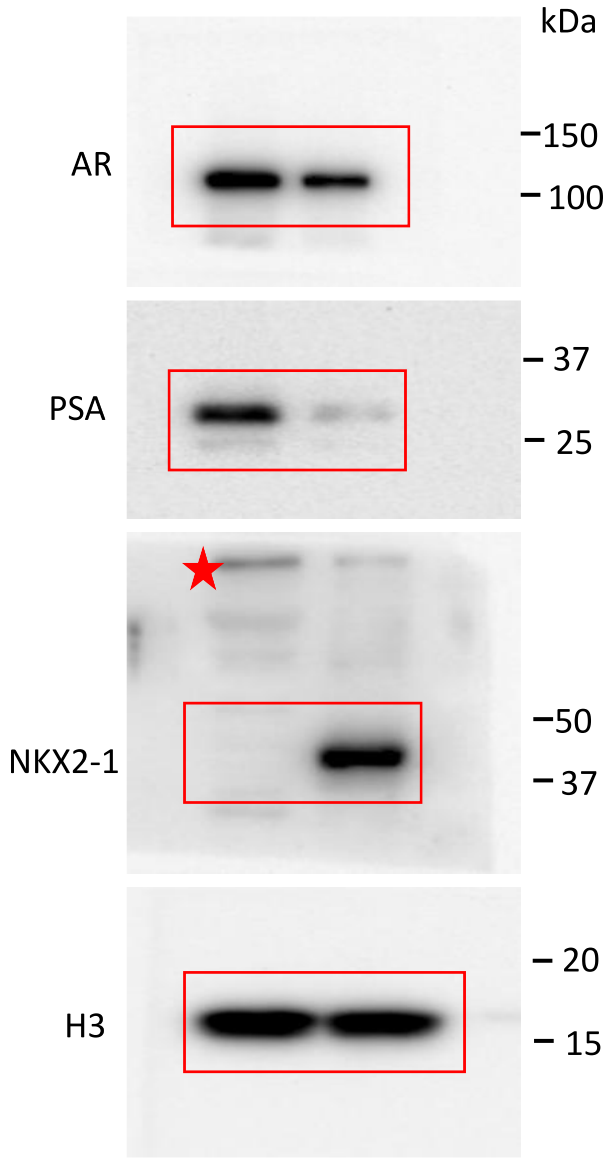


★ Non-specific band

Extended Data Fig.9g

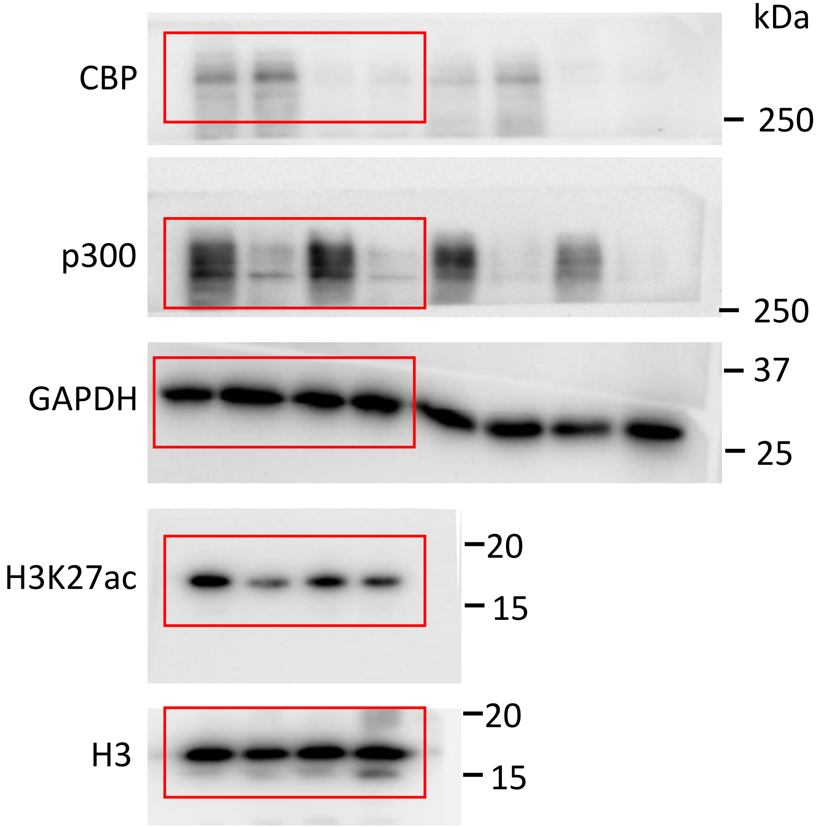

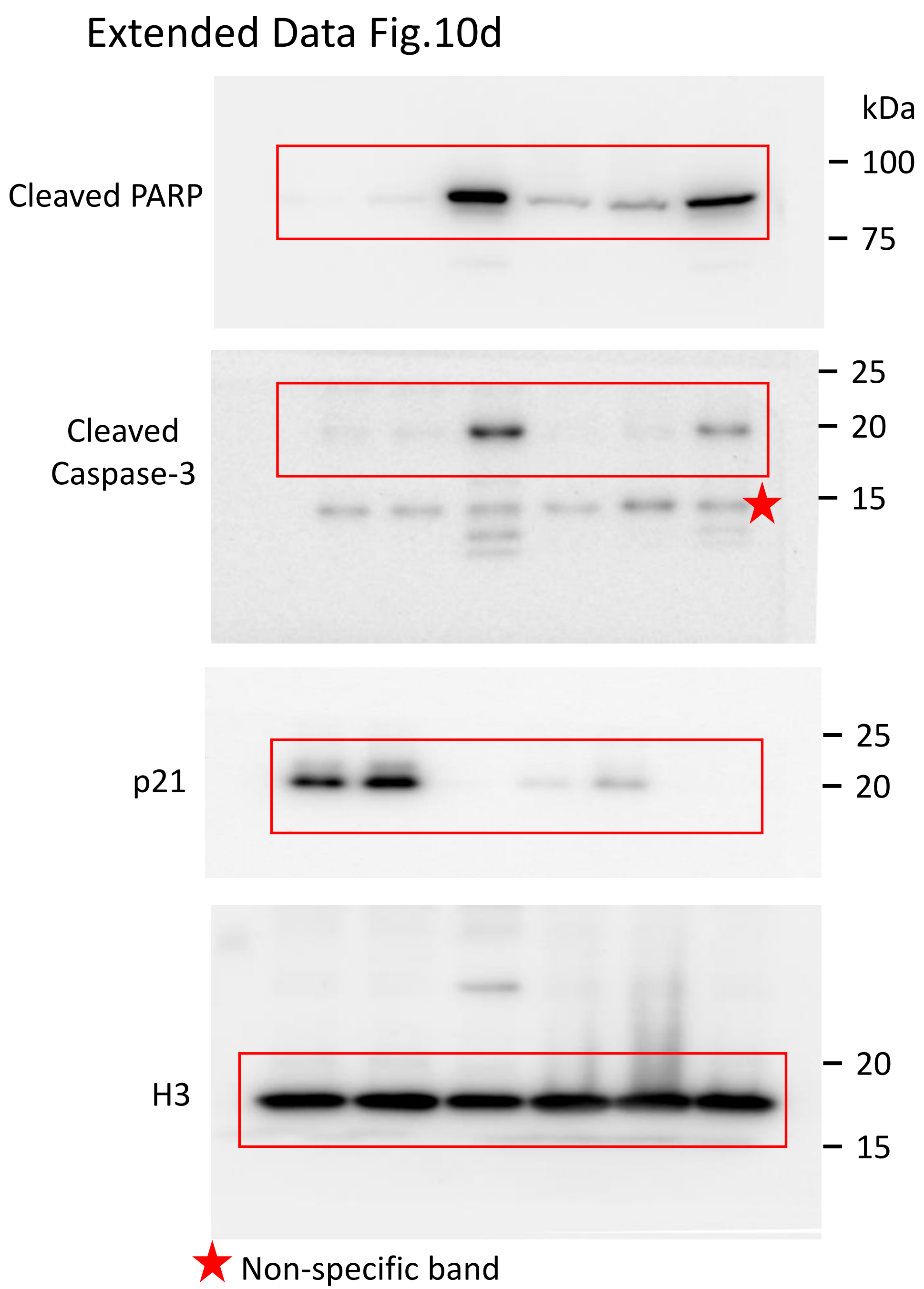
Extended Data Fig.10d
kDa
Cleaved PARP
100
75
Cleaved Caspase-3
25
20
15
p21
25
20
H3
20
15
Non-specific band

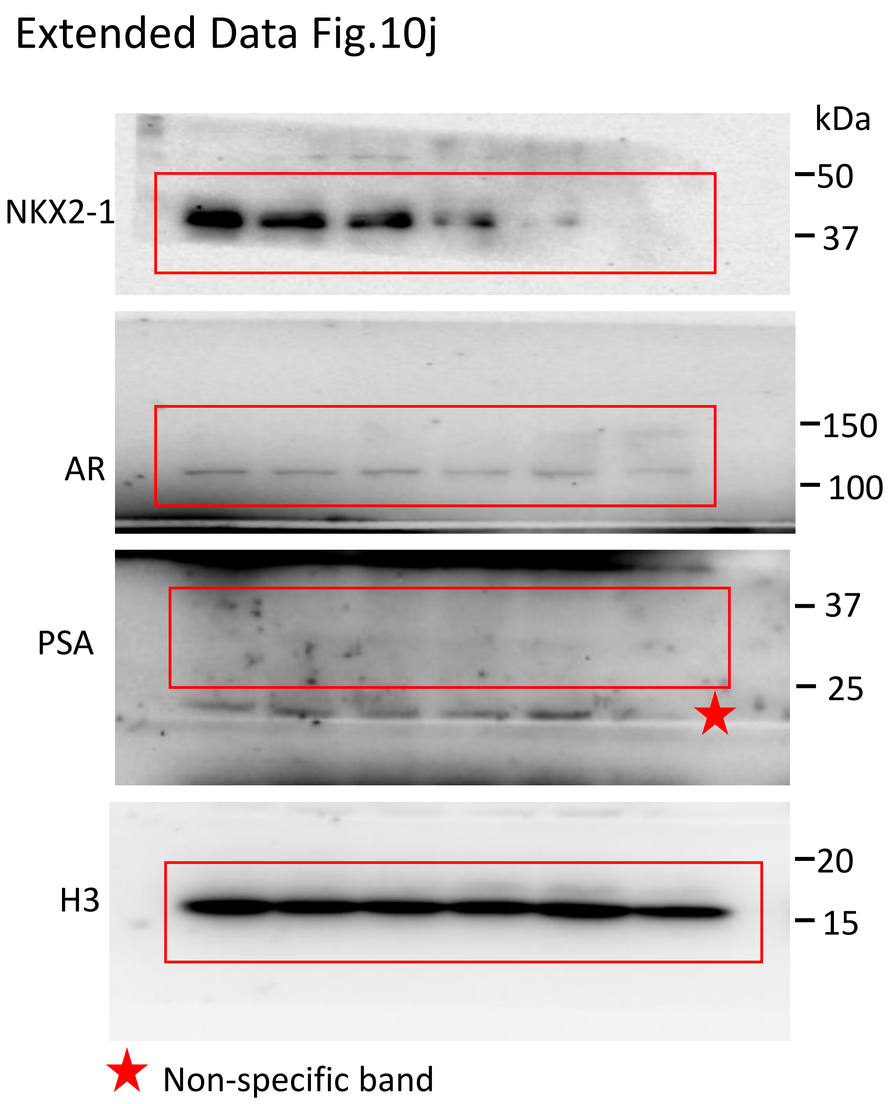
Extended Data Fig.10j
kDa
NKX2-1
50
37
AR
150
100
PSA
37
25
H3
20
15
Non-specific band
